# Mortality of Three Major Gynecological Cancers in the European Region: An Age–Period–Cohort Analysis from 1992 to 2021 and Predictions in a 25‑Year Period

**DOI:** 10.5334/aogh.4688

**Published:** 2025-06-10

**Authors:** Yanxue Lian, Pincheng Luo

**Affiliations:** 1 School of Medicine, University of Galway, Ireland

**Keywords:** gynecological cancers, mortality, European region, ovarian cancer, uterine cancer, cervical cancer

## Abstract

*Background:* The European region is marked by pronounced disparities in healthcare access, socioeconomic conditions, and cancer control policies, which influence the mortality trends of gynecological cancers across countries and may persist or intensify in the coming decades.

*Objective:* This study analyses mortality trends of three main gynecological cancers, including ovarian, uterine, and cervical cancers in the European region from 1992 to 2021 and projects rates for the next 25 years to support targeted public health interventions.

*Methods:* Data from the Global Burden of Disease 2021 were used. An age‑period‑cohort (APC) model estimated overall annual percentage changes in mortality (net drifts), local drifts, and age/period/cohort effects for gynecological cancers in the European region. A log‑linear APC model projected mortality and age‑standardized mortality rates (ASMRs) from 2022 to 2046.

*Findings:* Over the past three decades, the European region has had some of the highest mortality rates globally for ovarian and uterine cancers, while trends for cervical cancer have been more favorable. Overall, gynecological cancer mortality declined, though rates increased with age, but period and cohort effects weakened. Ovarian cancer mortality decreased in 17 of the 44 countries studied, while remaining stable in the others. Uterine cancer mortality rose in three countries, with the most pronounced increase observed in Italy. Cervical cancer mortality declined in 32 countries, with Italy being the only country to show an upward trend. Forecasts indicate a steady increase in uterine cancer deaths over the next 25 years, with slight decreases in ASMR, while ovarian cancer and cervical cancer deaths and ASMRs are projected to decline.

*Conclusion:* Despite overall progress in reducing gynecological cancer mortality, significant disparities remain, particularly among older populations and in certain countries such as Italy. Projections indicate a rise in uterine cancer mortality, highlighting the urgent need to strengthen early screening, preventive measures, and equitable healthcare strategies to reduce future disease burden.

## Introduction

Cervical cancer, uterine cancer, and ovarian cancer are the three primary gynecological cancers affecting the female reproductive system, which not only contribute substantially to cancer‑related mortality among women but also place a considerable strain on healthcare systems [1]. While these cancers differ in etiology, risk factors, and outcomes, they are all strongly influenced by contextual factors such as socioeconomic development, healthcare infrastructure, and national health policies, factors that shape prevention, early detection, and access to treatment [2–4].

Within this global landscape, the European region, comprising 44 countries across the Eastern, Central, and Western subregions and defined as one of the World Health Organization (WHO) regions in the Global Burden of Disease (GBD) 2021 [5], offers a particularly informative setting for studying temporal patterns in gynecological cancer mortality. The region includes countries with varying levels of development, from high to middle socio‑demographic Index (SDI), and shows clear differences in healthcare access and cancer control efforts between EU and non‑EU nations. Previous studies have shown that uterine and ovarian cancers tend to have a heavier burden in higher SDI regions, while cervical cancer has a heavier burden in lower SDI settings [6, 7]. Notably, several European countries have experienced substantial improvements in their SDI over the past three decades, highlighting a shifting epidemiological and developmental context that warrants updated analysis [8].

Europe’s heterogeneity is particularly evident in the prevention and management of cervical cancer. The EU has issued comprehensive guidelines promoting organized, population‑based screening programs with personal invitations and strict quality assurance measures [9]. Countries such as Finland, Sweden, and the Netherlands have implemented robust national programs aligned with these standards. However, others, like Romania and Bulgaria, still report low screening coverage or lack fully implemented programs [10]. Outside the EU, many countries rely on opportunistic screening, which often results in lower coverage and quality, exacerbated by limited infrastructure and funding [9]. For uterine and ovarian cancers, although effective population‑based screening does not exist, EU countries generally provide better access to early diagnosis and standardized treatment compared to their non‑EU counterparts [11].

Given these disparities in healthcare systems, policy implementation, and evolving socioeconomic conditions, the European region offers a valuable case for examining long‑term trends in gynecological cancer mortality. This study presents a comprehensive analysis of mortality trends for cervical, uterine, and ovarian cancers across 44 countries in this region from 1992 to 2021, with projections extending through 2046.

## Materials and Methods

GBD 2021 offers the latest comprehensive estimates of epidemiological data for 371 diseases and injuries across 204 countries and territories, spanning from 1990 to 2021 [8, 12]. Each recorded death in the GBD framework is assigned a single underlying cause, derived from a mutually exclusive and exhaustive list of diseases and injuries. Specific coding within the International Classification of Diseases and Injuries (ICD) was used to map deaths attributed to certain gynecological cancers. Deaths due to cervical cancer were identified using ICD codes C53–C53.9, D06–D06.9, D26.0 (ICD10). For uterine cancer, ICD codes C54–C54.9, D07.0–D07.2, D26.1–D26.9 (ICD10) were employed. Ovarian cancer deaths were mapped using ICD codes C56–C56.9, D27–D27.9, D39.1 (ICD10) [5, 8]. Detailed methodologies concerning data inputs, processing, synthesis, and final model development can be found in the GBD 2021 publications [5, 8].

The GBD study applies all available data sources across different age groups, timeframes, geographical regions, and health domains, leveraging standardized tools within a Bayesian analytical framework [5, 8]. This methodology ensures effective use of existing data, addressing challenges related to incomplete healthcare data access and supporting comprehensive global and regional disease burden estimates. This study was conducted in alignment with the ethical guidelines of the Declaration of Helsinki. Given that only de‑identified, aggregated data were utilized, the University of Washington Institutional Review Board approved a waiver of informed consent.

This study aimed to analyze mortality trends of three gynecological cancers, specifically ovarian cancer, uterine cancer, and cervical cancer, over a 30‑year period (1992–2021). Various metrics were examined, including the number of deaths, all‑age mortality rates (AAMRs), age‑standardized mortality rates (ASMRs), and relative percentage changes. These data points were directly extracted from GBD 2021, with all rates reported per 100,000 population. The 95% uncertainty intervals (UIs) were represented by the 25th and 975th values among 1,000 ordered estimates as per GBD algorithms [8]. Countries with limited data typically showed wider 95% UIs, reflecting lower accuracy in disease estimations [5].

### Analysis of overall temporal trends in the three gynecological cancers’ mortality

This study utilized a retrospective observational design. Temporal mortality trends from 1992 to 2021 were assessed using AAMRs and ASMRs, along with percentage changes in mortality rates. ASMR calculations employed global age‑standard population data from GBD 2021. Age distribution of deaths, as an indirect survival indicator, was evaluated by categorizing death counts into six age brackets (15–39, 40–49, 50–59, 60–69, 70–79, and 80+ for ovarian cancer and cervical cancer; 20–39, 40–49, 50–59, 60–69, 70–79, and 80+ for uterine cancer) and determining the proportion of deaths within each age group.

### Age–Period–Cohort modeling analysis of the three gynecological cancers’ mortality data

This study utilized the age–period–cohort (APC) model, a robust statistical method, to evaluate trends in mortality across different age groups, time periods, and birth cohorts [13]. The APC model has been extensively employed in epidemiological studies to detail long‑term mortality trends and to identify potential biological, societal, and technological influences on disease patterns [14, 15]. Structured as a log‑linear Poisson model, the APC analysis allows for estimating cumulative impacts over a Lexis diagram (see Supplementary Tables S1–3), which highlights variations by age, period, and cohort. Comprehensive explanations and methodological applications of the APC model are available in the existing literature [16, 17].

In this study, the APC model used population and mortality data related to gynecological cancers from various countries and territories for the period spanning 1992 to 2021. Data from GBD 2021 were structured in consistent five‑year intervals, with population and mortality records segmented into periods from 1992 to 1996 (median year: 1994) to 2017–2021 (median year: 2019). Age categories were similarly arranged in consecutive five‑year intervals, ranging from 15 to 19 years (for ovarian cancer and cervical cancer) or 20–24 years (for uterine cancer) up to 90–94 years. Birth years were grouped into five‑year intervals starting from 1900 to 1904 (median year: 1902) up to 1995–1999 (median year: 1997) for uterine cancer and 2000–2004 (median year: 2002) for ovarian cancer and cervical cancer.

The APC model assessed the combined impacts of age, period, and cohort effects on mortality trends, presented through net drift, which indicates the annual percentage change after adjusting for these variables [18]. Net drift reflects both period‑ and cohort‑specific trends, combining influences related to calendar time and generational effects [18]. The value of net drift is captured in the period (cohort) ratio curves. The selection of reference periods or cohorts was arbitrary and did not affect the interpretative conclusions of the analysis.

### Prediction modeling of the three gynecological cancers’ mortality data

Mortality rates were calculated for each five‑year age group across the entire population. ASMRs, expressed per 100,000 person‑years, were derived using global population and fertility projections [19]. To forecast mortality and ASMR for the period from 2022 to 2046 by country and age group, a log‑linear APC model was utilized, designed to moderate exponential growth, and restrict the linear projection of trends. This model, implemented through the NORDPRED package in R [20], has shown strong predictive performance for projecting cancer mortality trends [21, 22]. The model extrapolated data from the three or four most recent five‑year periods (depending on data availability) using a power function to level off the growth, while maintaining the linear trend of the past decade. Projections for the next periods were adjusted by 25%, 50%, and 75% in attenuation (or accentuation for negative trends). The projected death numbers in 2046 were derived by calculating a weighted average of the incidence rates from the final two prediction periods, centered on 2046, and applying these rates to national population projections for that year.

## Results

### Trends in mortality of three gynecological cancers in the European region, 1992–2021

Over the past 30 years, the global deaths from the three main gynecological cancers have risen. Uterine cancer saw a 72.5% increase in deaths, reaching 97.7 thousand in 2021. Ovarian cancer experienced a 76.3% rise to 185.6 thousand deaths, while cervical cancer increased by 38%, with 296.7 thousand deaths (Supplementary Table S4). Among the six WHO regions, the European region consistently reported the highest number of deaths from uterine cancer and ovarian cancer from 1992 to 2021, accounting for approximately one‑third of global cases (Supplementary Figures S1A, B). The European region also exhibited the highest AAMRs for both uterine cancer and ovarian cancer, nearly double that of the second highest region, the Americas. In terms of ASMRs, the European region saw decreases of 5.4% for uterine cancer and 19.9% for ovarian cancer in 2021 compared to 1992 levels (Table 1). Despite these reductions, the European region maintained the highest ASMR compared to other WHO regions. Conversely, mortality rates for cervical cancer in the European region have consistently declined over the past 30 years. In 2021, the European region ranked second to last in terms of cervical cancer death numbers, AAMRs, and ASMRs, only ahead of the Eastern Mediterranean region (Supplementary Figure S1C). Specifically, cervical cancer deaths in Europe decreased by 19.3%, reaching 30.7 thousand, which represented about one‑tenth of global cervical cancer deaths. Europe’s AAMR declined by 24.9%, a rate six times faster than the global average. Similarly, the decrease in ASMR in the European region outpaced the global decline.

**Table 1:** The trends in the three gynecological cancer mortality in the European region from 1992 to 2021.

	DEATH NUMBERS (*N*)			ALL‑AGE MORTALITY			AGE‑STANDARDIZED MORTALITY (PER 1,00,000)			
	Number in 1992	Number in 2021	Percent change of numbers, 1992–2021, %	Rate in 1992, per 1,00,000	Rate in 2021, per 1,00,000	Percent change of rate, 1992–2021, %	Rate in 1992	Rate in 2021	Percent change of rate, 1992–2021, %	Net drift of mortality, % per year
Uterine cancer	23,144 (21,964 to 23,951)	32,031 (28,735 to 34,200)	38.4	5.18 (4.92 to 5.36)	6.68 (5.99 to 7.14)	29	3.5 (3.33 to 3.61)	3.31 (3.02 to 3.52)	–5.4	–0.50 (–0.62 to −0.37)
Cervical cancer	37,986 (36,605 to 39,058)	30,653 (28,434 to 32,373)	–19.3	8.51 (8.2 to 8.75)	6.39 (5.93 to 6.75)	–24.9	6.31 (6.1 to 6.47)	3.82 (3.59 to 4.04)	–39.5	–1.78 (–1.89 to −1.67)
Ovarian cancer	46,292 (44,089 to 48,038)	53,324 (48,537 to 56,723)	15.2	10.37 (9.88 to 10.76)	11.12 (10.13 to 11.83)	7.2	7.33 (7.01 to 7.6)	5.87 (5.41 to 6.21)	–19.9	–0.97 (–1.05 to −0.89)

**Figure 1:**
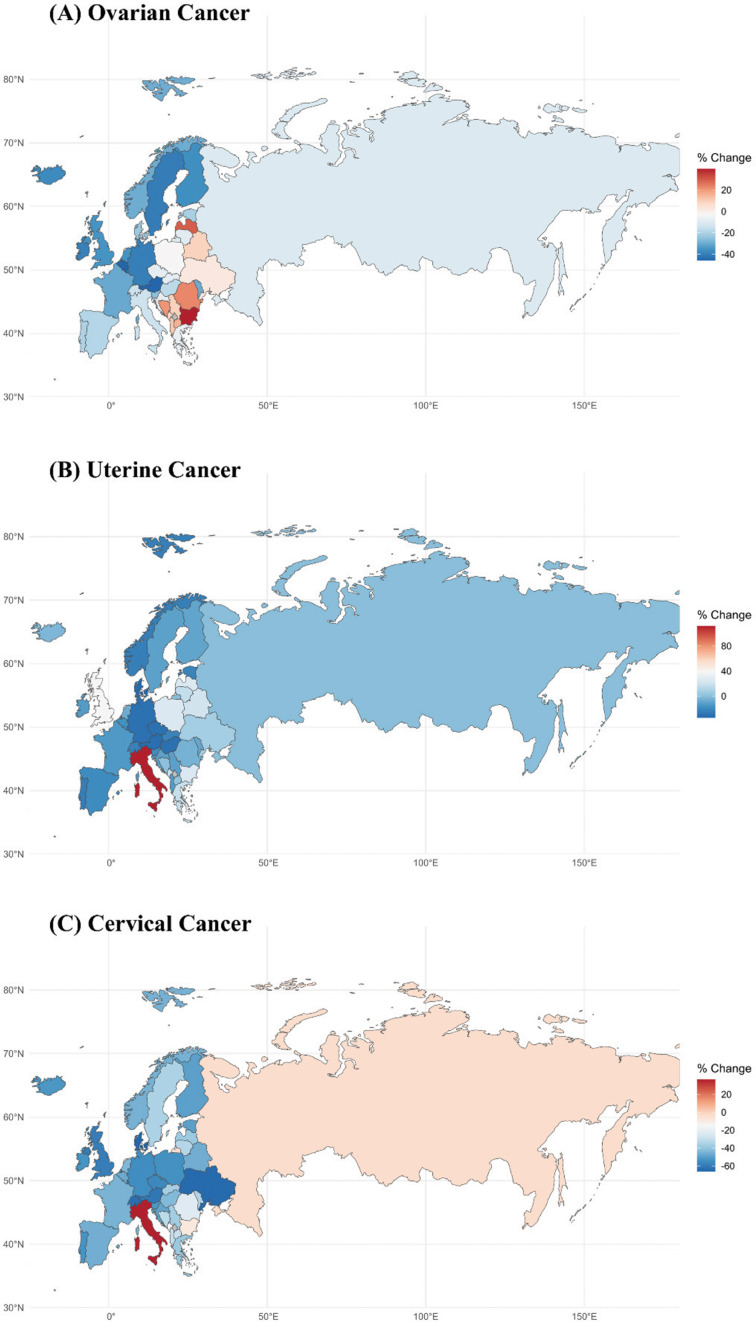
Maps of percent change in age‑standardized mortality rates for three gynecological cancers in the European region, 1992–2021.

Analyzing mortality trends across the 44 countries in the European region from 1992 to 2021 revealed that uterine cancer death numbers and AAMRs increased in most countries, though 33 countries saw a decrease in ASMRs. Italy experienced the largest increase in uterine cancer death numbers and AAMRs, rising by 228.2% and 211.9%, respectively. Italy also had the highest rise in ASMR, increasing by 112%, more than three times faster than the UK. Ovarian cancer mortality trends were similar, with Albania recording the highest increases in ovarian cancer deaths and AAMRs. Ten countries saw increases in ASMRs, with Bulgaria experiencing the greatest decrease (39.7%). In contrast, most European countries showed declines in cervical cancer deaths, AAMRs, and ASMRs. Ukraine had the greatest decrease in cervical cancer death numbers and AAMR, while Denmark recorded the largest reduction in ASMR (Figure 1, Supplementary Tables S5–7).

### Temporal trends in the three gynecological cancers’ mortality across age groups in the European region, 1992–2021

**Figure 2:**
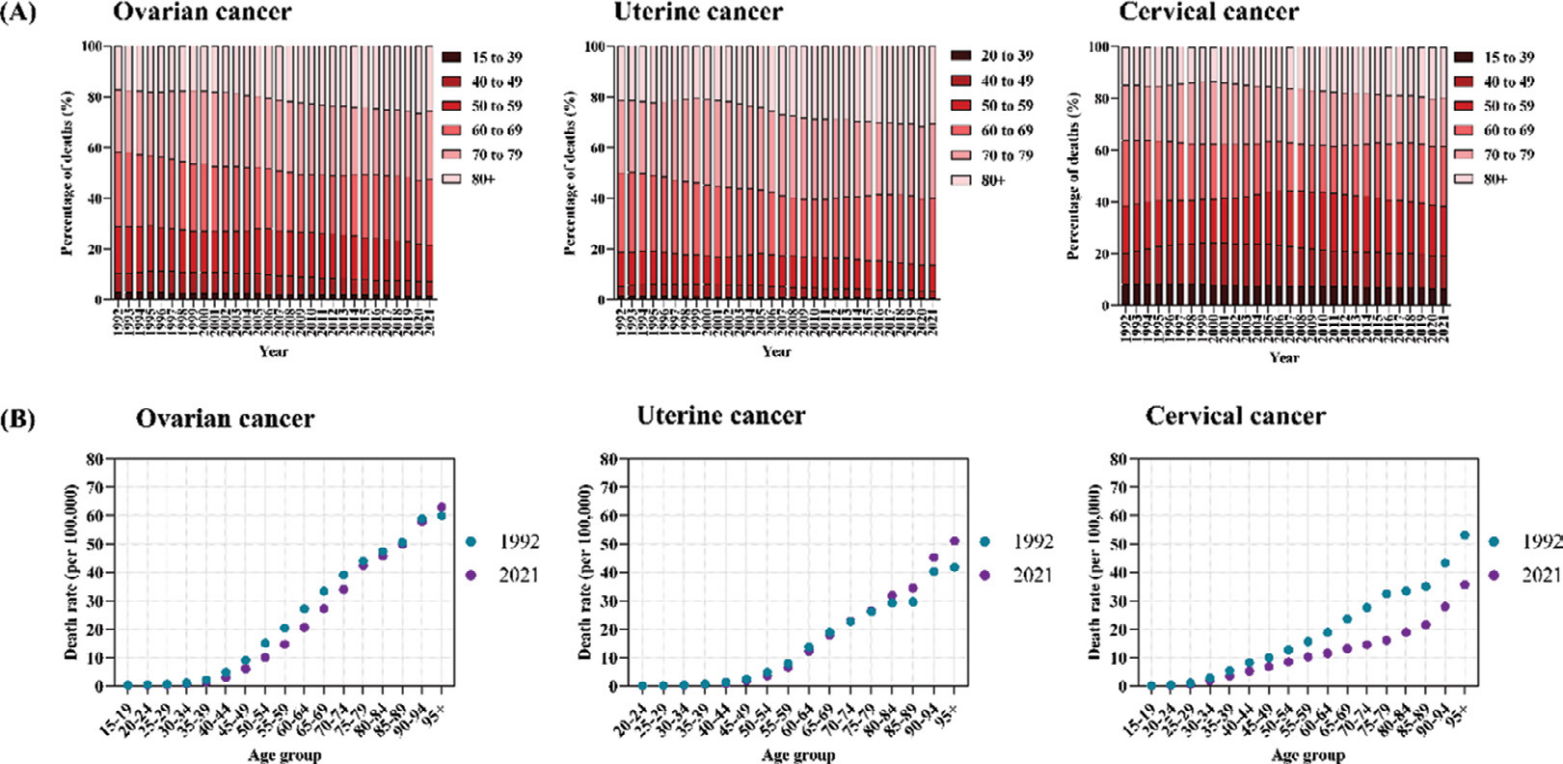
Temporal change in the mortality of three gynecological cancers across age groups in the European region, 1992–2021. (**A**) The relative percentage of deaths by age groups for three gynecological cancers; (**B**) the temporal changes in the mortality rate of three gynecological cancers by age groups.

The temporal trends in the age distribution of gynecological cancers‑related mortality in the European region, as illustrated in Figure 2A, provide an indirect measure of population survival rates. Over the past three decades, individuals aged 60 and above have consistently accounted for more than 70% of overall mortality due to ovarian cancer and uterine cancer. In contrast, cervical cancer mortality has been more evenly distributed across age groups, with individuals under 60 years contributing to approximately half of cervical cancer deaths, and the 15–39 age group alone representing about 10% of cervical cancer mortality. Notably, a gradual shift in mortality from all three gynecological cancers towards older age groups (60–69, 70–79, and 80+ years) has been observed across the European region, with few exceptions such as San Marino. This shift reflects the increasing impact of these cancers on an aging population (Supplementary Figures S2–4).

Over the period from 1992 to 2021, the mortality rates of uterine cancer and ovarian cancer in the European region have shown contrasting trends across age groups. While younger populations experienced a decline, rates increased among older individuals. For those aged 95 and above, the ovarian cancer mortality rate rose by 5.0% during this 30‑year span. Similarly, uterine cancer mortality rates in individuals aged 80 and older were higher in 2021 compared to 1992, with the most pronounced increases occurring in the oldest age groups. These trends were reflected in many countries within the European region (Supplementary Figures S5 and S6). In contrast, cervical cancer mortality rate decreased across all age groups in the European region by 2021 compared to 1992 (Figure 2B). However, exceptions to this trend of cervical cancer were also observed in countries such as Albania, Bulgaria, Cyprus, Greece, Italy, Malta, Montenegro, North Macedonia, Norway, Poland, Romania, the Russian Federation, Slovakia, Slovenia, Sweden, and Switzerland (Supplementary Figure S7).

### Local drift, age, period, and cohort effects on the three gynecological cancers in the European region

**Figure 3:**
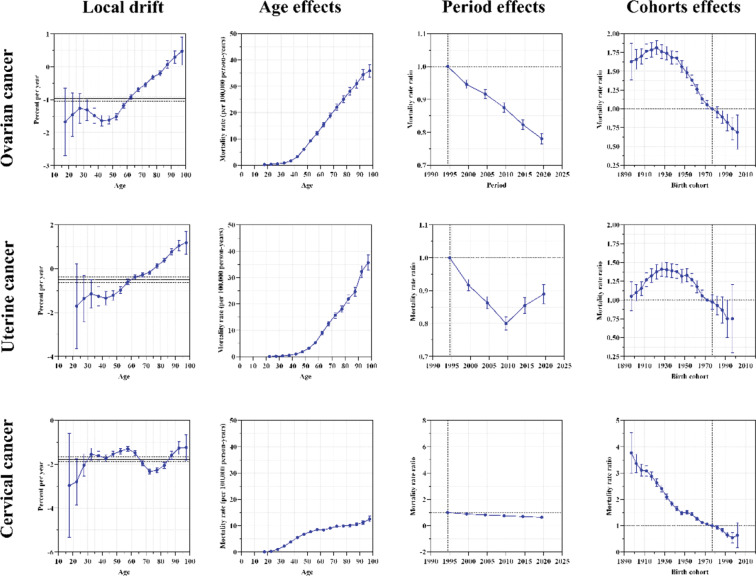
The local drifts, age effects, period effects, and cohort effects of three gynecological cancers‑related mortality in the European region from 1992 to 2021.

Figure 3 provides key insights into the metrics derived from the APC model, which includes local drift (the annual percentage change in mortality rates by age group), net drift (the overall annual change across all ages), age effects (represented by the longitudinal age curve capturing natural variations in mortality), period effects (illustrating the relative risk of mortality across time periods to assess progress), and cohort effects (depicting the relative risk of mortality across birth cohorts). These measures are presented for three common gynecological cancers: ovarian cancer, cervical cancer, and uterine cancer.

The net drift of ovarian cancer‑related mortality in the European region demonstrated the largest decline among all six WHO regions, at −0.97% (95% CI: −1.05% to −0.89%), surpassing the global rate of −0.53% (95% CI: −0.58% to −0.48%) (Supplementary Table S4). The local drift revealed an increasing trend among individuals aged over 85 years, while those younger than 85 exhibited a declining trend (Figure 3). Similarly, the European region showed the steepest decline in the net drift of cervical cancer, compared to the other WHO regions, although the global rate stood at −1.17% (95% CI: −1.23% to −1.12%). The local drift of cervical cancer indicated a downward trend across all age groups. In contrast, the net drift of uterine cancer mortality in the European region, at −0.50% (95% CI: −0.62% to −0.37%) per year, was not only lower than the global rate of −0.98% (95% CI: −1.03% to −0.92%) per year, but also lower than that observed in the Western Pacific region [−1.99% (95% CI: −2.09% to −1.89%) per year] and the South‑East Asia region [−0.51% (95% CI: −0.68% to −0.35%) per year]. The local drift for uterine cancer revealed an upward trend in individuals over 75 years of age, while for those under 75, the trend was declining.

The longitudinal age patterns showed a progressive increase in mortality with age for all three gynecological cancers. For ovarian cancer and uterine cancer, the mortality rates increased slowly in the younger age groups but escalated rapidly in the older age groups, with age groups 40–44 and 50–54 marking the turning points, respectively. However, the pattern for cervical cancer was distinct: mortality increased gradually up to the age group 50–54, after which the rate of increase became more moderate. Regarding period effects, a sharp decline in ovarian cancer‑related mortality was observed, accompanied by a more modest reduction in cervical cancer‑related mortality. For uterine cancer, the period effects initially demonstrated a decreasing trend, followed by a marked rise beginning in 2012. Finally, the cohort effects indicated a positive reduction in mortality across all three gynecological cancers, underscoring improvements across successive birth cohorts.

### APC effects on the three gynecological cancers in the countries of European region

Local drift, as well as age, period, and cohort effects, varied across countries in the European region (Supplementary Figures S8–19). For uterine cancer, a positive net drift was observed in three countries: Bulgaria, Italy, and the United Kingdom, with Italy showing the highest net drift at 2.89%. Conversely, three countries, Germany, Hungary, and the Russian Federation, showed a negative net drift (Supplementary Table S2 and Supplementary Figure S12). For ovarian cancer, 17 out of 44 countries exhibited a negative net drift, while the remaining countries showed a relatively stable drift, with the 95% confidence interval including 0 (Supplementary Table S3 and Supplementary Figure S8). In the case of cervical cancer, 32 countries experienced a negative net drift, with Italy being the only country showing a positive net drift among the 44 European countries analyzed (Supplementary Table S4 and Supplementary Figure S16).

After adjusting for period and cohort effects, age effects indicated that mortality rates for all three gynecological cancers rose with age, particularly after 40 years. Ovarian cancer mortality peaked in the 90–94 age group, followed by a sharp decline in those aged 95 and above, a trend observed in 19 countries. However, in Austria, Belgium, Czechia, Denmark, Finland, Germany, Hungary, Iceland, Ireland, the Netherlands, Norway, the Russian Federation, Slovakia, Switzerland, and Ukraine, the ovarian cancer mortality peak occurred at younger ages (65–89 years; Supplementary Figure S9). For cervical cancer, the age effects on mortality rates showed a similar trend, with a younger peak observed in Czechia, Hungary, the Netherlands, Poland, the Republic of Moldova, Romania, the Russian Federation, and Ukraine (Supplementary Figure S17). Conversely, uterine cancer mortality rates consistently increased with age across the European region, with the highest mortality in the 95‑and‑above age group (Supplementary Figure 13).

Positive trends were also observed in the period and cohort effects for ovarian cancer and cervical cancer. Specifically, period effects for ovarian cancer decreased in 30 countries, while cohort effects decreased in 31 countries (Supplementary Figures S10 and S11). For cervical cancer, increased period effects were limited to six countries, Andorra, Bulgaria, Italy, Monaco, Montenegro, and San Marino, whereas cohort effects decreased for cervical cancer across all countries in the region (Supplementary Figures S18 and S19). In contrast, trends for uterine cancer were less favorable; increased period effects were observed in about half of the countries, and cohort effects rose in 32 countries, suggesting growing concerns in the region (Supplementary Figures S10 and S15).

### Trends in the three gynecological cancers’ mortality forecast, 2022–2046

**Figure 4:**
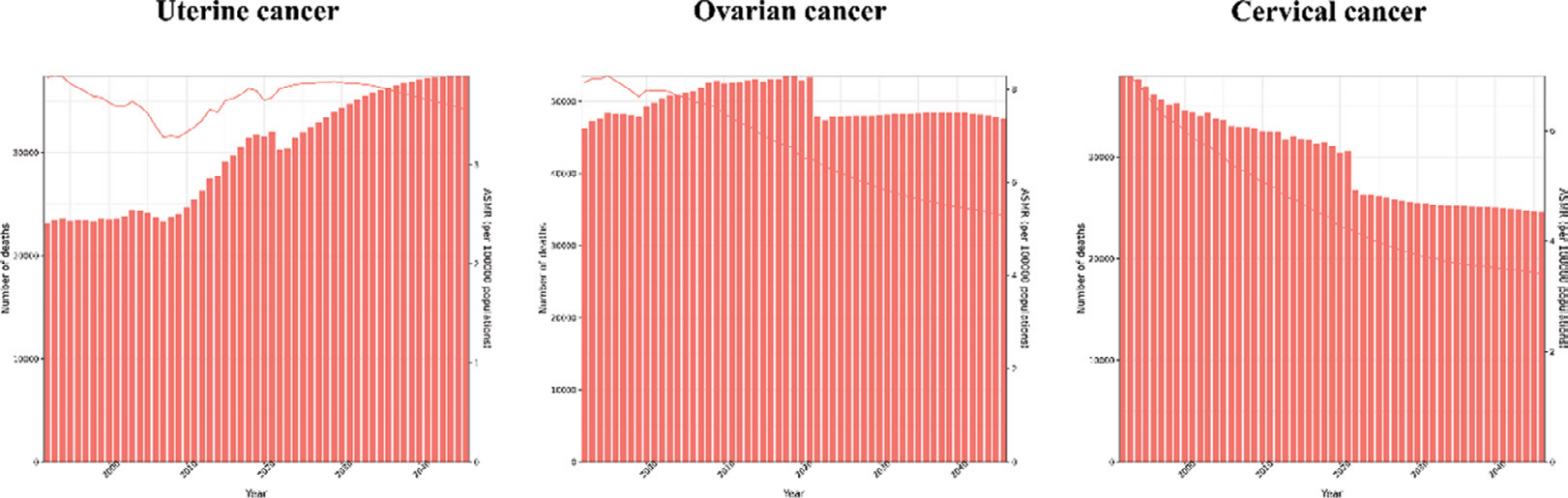
Observed and predicted death numbers and age‑standardized mortality rates related to three gynecological cancers in European region from 1992 to 2046.

Forecasts of mortality numbers and ASMRs for the three gynecological cancers in the European region are illustrated in Figure 4. Despite a projected slight decrease in ASMR, the number of deaths due to uterine cancer is expected to rise significantly, from 32,031 in 2021 to 37,465 in 2046 across the European region. This trend may be driven by increased uterine cancer mortality in 37 out of 42 countries, with the exception of Bulgaria, Estonia, Latvia, Lithuania, and Serbia. Eight countries are also forecasted to have higher ASMRs for uterine cancer in 2046 compared to 2021 (Supplementary Figure S21).

Encouraging trends are noted in the forecasts for ovarian cancer and cervical cancer mortality. Ovarian cancer‑related deaths are projected to decline in the European region, from 53,324 in 2021 to 47,643 in 2046, though notable increases in ovarian cancer mortality are anticipated in certain countries, such as Albania, Andorra, Cyprus, Greece, Iceland, Ireland, Israel, Luxembourg, North Macedonia, Portugal, Slovakia, and Spain. ASMR for ovarian cancer is projected to decrease both regionally and within all countries analyzed (Supplementary Figure S20).

Similarly, forecasts indicate a decline in cervical cancer mortality to 24,594 cases, with an ASMR of 3.42 per 100,000 by 2046 in the European region. However, compared to 2021, 19 countries are expected to experience higher cervical cancer‑related mortality by 2046, including Albania, Andorra, Austria, Belgium, Cyprus, Finland, France, Greece, Iceland, Israel, Italy, Luxembourg, the Netherlands, Norway, the Republic of Moldova, the Russian Federation, Spain, Sweden, and the United Kingdom. Nonetheless, all countries in the region are anticipated to observe a reduction in ASMR for cervical cancer (Supplementary Figure S22).

## Discussion

This study is a comprehensive analysis of the uterine cancer, ovarian cancer and cervical cancer mortality burdens in the European region under the APC framework. For three decades, the European region has consistently led the world in both ovarian cancer and uterine cancer mortality burden, ranking first among the six WHO regions, a troubling public health issue, while cervical cancer in the region is relatively optimistic. Generally, although the European region showed promising overall declines in all three gynecological cancers’ mortality, with mortality increasing with age and weakening period and cohort effects, certain countries deviate markedly, such as Italy, highlighting disparities in healthcare resources and suggesting a need for targeted interventions. These findings underscore the importance of enhancing access to early screening and prevention in specific regions to address the unique challenges posed by gynecological cancer mortality across different European populations. The forecast results have shown that the number of uterine cancer deaths will increase steadily in the next 25 years, with slightly decreased ASMR, while the death numbers and ASMR of ovarian cancer and cervical cancer dramatically decrease.

### Ovarian cancer

A general decline of ovarian cancer mortality rates in the European region was likely to contribute to the decrease in period and cohort effects, which reflected a combination of medical advances, public health efforts, and generational changes. Period effects have likely declined due to improvements in diagnostic imaging (CT, ultrasound, MRI) [23], greater public awareness [23], reduced hormone replacement therapy use since the early 2000s, and the introduction of more effective treatments such as platinum‑based chemotherapy, taxanes, and PARP inhibitors [24]. Cohort effects suggested that generational changes, particularly the widespread use of oral contraceptives, which reduce ovarian cancer risk by up to 50%, have contributed significantly to declining mortality [24, 25]. Additionally, shifts in reproductive behaviors, healthier lifestyles, and improved health awareness in younger cohorts have further supported this downward trend [26].

Although ovarian cancer mortality rates have shown an overall annual decline, the European region still experienced the highest mortality burden among all WHO regions throughout the study period. Several factors may help explain this pattern. The European region is one of the most rapidly aging regions in the world, with over one‑fifth of its population aged 65 and older, and a steadily rising median age [27]. Given that age has a strong influence on ovarian cancer outcomes, as reflected in our age effect analysis, which showed a sharp rise in mortality beginning around perimenopause or early menopause, this demographic shift likely contributes to the higher mortality. Population aging may also account for the observed increase in mortality rates among individuals aged over 95 in 2021 relative to 1992.

Not only that, the European region also reported the highest incidence of ovarian cancer globally [28], which naturally contributes to a higher mortality rate. Aging is a major risk factor, with studies suggesting that 83% to 97% of ovarian cancer cases occur in women over 40 [29, 30]. Older patients often present with advanced stages of ovarian cancer at diagnosis [31], which is compounded by the absence of standardized screening programs [32]. Advanced age also correlates with higher tumor grades and more comorbidities, factors that can complicate treatment options and outcomes [25]. Reduced immune function and increased tumor aggressiveness in older populations further contribute to higher mortality rates [25]. Lastly, genetic predisposition may play a role, particularly in Eastern Europe, where women have been reported to carry a relatively high risk of hereditary ovarian cancer [33].

### Uterine cancer

As with ovarian cancer, advances in medicine, public health initiatives, and generational shifts have contributed to slowing the annual percentage increase in uterine cancer mortality. However, these gains have been outweighed by the growing impact of population aging and the region’s high incidence of uterine cancer [34], resulting in the highest uterine cancer mortality burden among all WHO regions. The age effects observed for uterine cancer mirror those of ovarian cancer, with mortality rates rising sharply around the time of perimenopause or early menopause (approximately age 50). This acceleration may be driven by similar factors, such as higher incidence rates in older women [35, 36], more frequent late‑stage diagnoses [37], the absence of standardized screening programs [32], more aggressive tumor biology, and age‑related declines in immune function [25].

Despite regional trends pointing toward improved uterine cancer outcomes, three countries showed increasing mortality rates over the 30‑year study period, with Italy experiencing the most notable rise. This was driven by concurrent increases in age, period, and cohort effects. Italy has one of the oldest populations in the world and holds the highest median age in the EU [27]. The rising cohort effects were likely partially driven by long‑term changes in reproductive behavior. Italy reported some of the highest rates of nulliparity and delayed childbearing in Europe [38], both established risk factors for endometrial cancer [2].

Additionally, Italy’s decentralized National Health Service has resulted in geographic disparities in access to cancer diagnosis and treatment, particularly between the northern and southern regions [39]. Compounding this, the economic recession of the early 1990s led to cuts in public health funding, which may have disrupted preventive services and delayed the adoption of updated cancer management protocols. Together, these demographic, structural, and policy‑related factors likely contributed to the upward trends in uterine cancer mortality observed in Italy over the study period.

### Cervical cancer

Unlike ovarian and uterine cancers, the age effect for cervical cancer shows a distinct pattern: mortality increases relatively quickly among women under 50, but more gradually in older age groups, though the overall mortality burden still rises with age. This may be due to a higher incidence of aggressive cervical cancer types in younger women [40], who are often diagnosed at more advanced stages. Younger women may present with non‑squamous histology, higher rates of distant metastases, and poorer prognosis [41]. Furthermore, participation in cervical cancer screening programs among young women in many developed countries has been slowly declining in recent years for unknown reasons [42]. Although standardized cervical cancer screening is available, discontinuing screening after age 60 or 65 is suggested in most European countries if they have either three consecutive negative cytology tests within the last 10 years or two consecutive negative HPV tests, and if they have no history of cervical intraepithelial neoplasia grade 2 or more severe disease in the past 25 years [9]. This can lead to lower screening rates in older women, who may falsely believe they are no longer at risk.

Italy was the only country in the study to show an upward trend in cervical cancer mortality over the observation period, while both the incidence and mortality rates in Italy remained below the European average. The observed rise in mortality appears to be largely driven by increasing period effects, consistent with findings from previous research [43]. This trend may reflect shortcomings in the effectiveness of cervical cancer screening and early detection strategies [43]. Nationally, participation in organized screening programs remains suboptimal, with reported compliance rates ranging from 32% to 41% [44]. A substantial proportion of screening continues to occur opportunistically, outside organized programs. Such opportunistic screening is less effective, as it often fails to reach women at highest risk and lacks standardized follow‑up and quality control.

Additionally, despite the inclusion of HPV vaccination in the National Immunization Plan, coverage has declined [45]. Significant regional heterogeneity regarding the organizational and operational aspects of HPV vaccination and cervical cancer screening, with higher mortality rates, was observed in Southern Italy compared to Northern regions due to disparities in healthcare access and quality [45]. Enhancing HPV vaccination coverage and screening initiatives could reduce cervical cancer mortality in these countries.

## Conclusion

This study highlights significant progress in reducing mortality rates for all three gynecological cancers, especially cervical cancer, in the European region, despite persistent disparities among countries. While overall trends show declining mortality over the studied 30 years, particularly due to advances in diagnostic technologies, public health initiatives, and preventive strategies such as increased contraceptive use and HPV vaccination, challenges remain, especially among older populations and specific nations with rising period and cohort effects. Projections indicate a rise in gynecological cancer deaths across Europe over the next 25 years, particularly due to uterine cancer. Strengthening early screening, prevention, and equitable healthcare is essential to mitigate this future burden.

## Data Availability

The data that support the findings of this study are available in the Global Burden of Disease website at https://ghdx.healthdata.org/gbd-2021 (Institute for Health Metrics and Evaluation, 2024). These data were derived from the following resources available in the public domain: https://ghdx.healthdata.org/gbd-2021.

## Additional Files

The additional files for this article can be found as follows:

Supplementary Table 1. The Lexis diagram of GBD 2021 data used for the APC model of ovarian cancer mortality in the European Region.

Supplementary Table 2. The Lexis diagram of GBD 2021 data used for the APC model of uterine cancer mortality in the European Region.

Supplementary Table 3. The Lexis diagram of GBD 2021 data used for the APC model of cervical cancer mortality in the European Region.

Supplementary Table 4. The global trends in the mortality of the three gynecological cancers (ovarian, uterine, and cervical cancers) from 1992 to 2021.

Supplementary Table 5. The mortality of ovarian cancer in all countries of the European Region from 1992 to 2021.

Supplementary Table 6. The mortality of uterine cancer in all countries of the European Region from 1992 to 2021.

Supplementary Table 7. The mortality of cervical cancer in all countries of the European Region from 1992 to 2021.

Supplementary Figure 1. Temporal trends of the numbers of death, all‑age mortality, and age‑standardized mortality for three gynecological cancers (ovarian, uterine, and cervical cancers) among the six WHO regions from 1992 to 2021.

Supplementary Figure 2. The temporal change in the relative percentage of ovarian cancer deaths across different age groups in 44 countries within the European Region from 1992 to 2021.

Supplementary Figure 3. The temporal change in the relative percentage of uterine cancer deaths across different age groups in 44 countries within the European Region from 1992 to 2021.

Supplementary Figure 4. The temporal change in the relative percentage of cervical cancer deaths across different age groups in 44 countries within the European Region from 1992 to 2021.

Supplementary Figure 5. The temporal change of the death rate for ovarian cancer across age groups in 44 countries within the European Region from 1992 to 2021.

Supplementary Figure 6. The temporal change of the death rate for uterine cancer across age groups in 44 countries within the European Region from 1992 to 2021.

Supplementary Figure 7. The temporal change of the death rate for cervical cancer across age groups in 44 countries within the European Region from 1992 to 2021.

Supplementary Figure 8. The local drift of ovarian cancer deaths in 44 countries within the European Region from 1992 to 2021.

Supplementary Figure 9. The age effects of ovarian cancer deaths in 44 countries within the European Region from 1992 to 2021.

Supplementary Figure 10. The period effects of ovarian cancer deaths in 44 countries within the European Region from 1992 to 2021.

Supplementary Figure 11. The cohort effects of ovarian cancer deaths in 44 countries within the European Region from 1992 to 2021.

Supplementary Figure 12. The local drift of uterine cancer deaths in 44 countries within the European Region from 1992 to 2021.

Supplementary Figure 13. The age effects of uterine cancer deaths in 44 countries within the European Region from 1992 to 2021.

Supplementary Figure 14. The period effects of uterine cancer deaths in 44 countries within the European Region from 1992 to 2021.

Supplementary Figure 15. The cohort effects of uterine cancer deaths in 44 countries within the European Region from 1992 to 2021.

Supplementary Figure 16. The local drift of cervical cancer deaths in 44 countries within the European Region from 1992 to 2021.

Supplementary Figure 17. The age effects of cervical cancer deaths in 44 countries within the European Region from 1992 to 2021.

Supplementary Figure 18. The period effects of cervical cancer deaths in 44 countries within the European Region from 1992 to 2021.

Supplementary Figure 19. The cohort effects of cervical cancer deaths in 44 countries within the European Region from 1992 to 2021.

Supplementary Figure 20. Observed and predicted ovarian cancer‑related death numbers and age‑standardized mortality rate in 44 countries within the European Region from 1992 to 2046.

Supplementary Figure 21. Observed and predicted uterine cancer‑related death numbers and age‑standardized mortality rate in 44 countries within the European Region from 1992 to 2046.

Supplementary Figure 22. Observed and predicted cervical cancer‑related death numbers and age‑standardized mortality rate in 44 countries within the European Region from 1992 to 2046.

## References

[r1] Siegel RL, Miller KD, Fuchs HE, Jemal A. Cancer statistics, 2022. CA Cancer J Clin. 2022;72(1):7–33. 35020204 10.3322/caac.21708

[r2] Katagiri R, Iwasaki M, Abe SK, et al. Reproductive factors and endometrial cancer risk among women. JAMA Netw Open. 2023;6(9):e2332296. 37669051 10.1001/jamanetworkopen.2023.32296 PMC10481237

[r3] Stelzle D, Tanaka LF, Lee KK, et al. Estimates of the global burden of cervical cancer associated with HIV. Lancet Glob Health. 2021;9(2):e161–e169. 33212031 10.1016/S2214-109X(20)30459-9 PMC7815633

[r4] Webb PM, Jordan SJ. Global epidemiology of epithelial ovarian cancer. Nat Rev Clin Oncol. 2024;21(5):389–400. 38548868 10.1038/s41571-024-00881-3

[r5] Naghavi M, Ong KL, Aali A, et al. Global burden of 288 causes of death and life expectancy decomposition in 204 countries and territories and 811 subnational locations, 1990–2021: A systematic analysis for the Global Burden of Disease Study 2021. Lancet. 2024;403(10440):2100–2132. 38582094 10.1016/S0140-6736(24)00367-2 PMC11126520

[r6] Liu Y, Shi W, Mubarik S, Wang F. Assessment of secular trends of three major gynecologic cancers burden and attributable risk factors from 1990 to 2019: An age period cohort analysis. BMC Public Health. 2024;24(1):1349. 38764017 10.1186/s12889-024-18858-3 PMC11103856

[r7] Yang L, Yuan Y, Zhu R, Zhang X. Time trend of global uterine cancer burden: An age‑period‑cohort analysis from 1990 to 2019 and predictions in a 25‑year period. BMC Womens Health. 2023;23(1):384. 37480027 10.1186/s12905-023-02535-5 PMC10362563

[r8] Ferrari AJ, Santomauro DF, Aali A, et al. Global incidence, prevalence, years lived with disability (YLDs), disability‑adjusted life‑years (DALYs), and healthy life expectancy (HALE) for 371 diseases and injuries in 204 countries and territories and 811 subnational locations, 1990–2021: A systematic analysis for the Global Burden of Disease Study 2021. Lancet. 2024;403(10440):2133–2161. 38642570 10.1016/S0140-6736(24)00757-8 PMC11122111

[r9] Arbyn M, Anttila A, Jordan J, et al. European guidelines for quality assurance in cervical cancer screening. Second edition—summary document. Ann Oncol. 2010;21(3):448–458. 20176693 10.1093/annonc/mdp471 PMC2826099

[r10] Bøje RB, Bardou M, Mensah K, et al. What are the barriers towards cervical cancer screening for vulnerable women?: A qualitative comparative analysis of stakeholder perspectives in seven European countries. BMJ Open. 2024;14(5):e079921. 10.1136/bmjopen-2023-079921 PMC11103196 38760040

[r11] Pousette A, Hofmarcher T. Tackling inequalities in cancer care in the European Union. European Federation of Pharmaceutical Industries and Associations. 2024.

[r12] Institute for Health Metrics and Evaluation. Global Burden of Disease Study 2021 (GBD 2021) data resources. Seattle: IHME; 2024.

[r13] Rosenberg PS, Anderson WF. Age‑period‑cohort models in cancer surveillance research: Ready for prime time? Cancer Epidemiol Biomarkers Prev. 2011;20(7):1263–1268. 21610223 10.1158/1055-9965.EPI-11-0421 PMC3132831

[r14] Falcaro M, Castañon A, Ndlela B, et al. The effects of the national HPV vaccination programme in England, UK, on cervical cancer and grade 3 cervical intraepithelial neoplasia incidence: A register‑based observational study. Lancet. 2021;398(10316):2084–2092. 34741816 10.1016/S0140-6736(21)02178-4

[r15] Zou Z, Cini K, Dong B, et al. Time trends in cardiovascular disease mortality across the BRICS. Circulation. 2020;141(10):790–799. 31941371 10.1161/CIRCULATIONAHA.119.042864

[r16] Liu Z, Su Z, Li W, et al. Global, regional, and national time trends in disability‑adjusted life years, mortality, and variable risk factors of non‑rheumatic calcified aortic valve disease, 1990–2019: An age‑period‑cohort analysis of the Global Burden of Disease 2019 study. J Thorac Dis. 2023;15(4):2079–2097. 37197484 10.21037/jtd-23-480 PMC10183530

[r17] Su Z, Zou Z, Hay SI, et al. Global, regional, and national time trends in mortality for congenital heart disease, 1990–2019: An age‑period‑cohort analysis for the Global Burden of Disease 2019 study. EClinicalMedicine. 2022;43:101249. 35059612 10.1016/j.eclinm.2021.101249 PMC8760503

[r18] Rosenberg PS, Check DP, Anderson WF. A web tool for age–period–cohort analysis of cancer incidence and mortality rates. Cancer Epidemiol Biomarkers Prev. 2014;23(11):2296–2302. 25146089 10.1158/1055-9965.EPI-14-0300 PMC4221491

[r19] Vollset SE, Goren E, Yuan CW, et al. Fertility, mortality, migration, and population scenarios for 195 countries and territories from 2017 to 2100: A forecasting analysis for the Global Burden of Disease Study. Lancet. 2020;396(10258):1285–1306. 32679112 10.1016/S0140-6736(20)30677-2 PMC7561721

[r20] Møller B, Fekjær H, Hakulinen T, et al. Prediction of cancer incidence in the Nordic countries: Empirical comparison of different approaches. Stat Med. 2003;22(17):2751–2766. 12939784 10.1002/sim.1481

[r21] Ferlay J, Steliarova‑Foucher E, Lortet‑Tieulent J, et al. Cancer incidence and mortality patterns in Europe: Estimates for 40 countries in 2012. Eur J Cancer. 2013;49(6):1374–1403. 23485231 10.1016/j.ejca.2012.12.027

[r22] Olsen AH, Parkin DM, Sasieni P. Cancer mortality in the United Kingdom: Projections to the year 2025. Br J Cancer. 2008;99(9):1549–1554. 18854832 10.1038/sj.bjc.6604710 PMC2579704

[r23] La Vecchia C, Rota M, Malvezzi M, Negri E. Potential for improvement in cancer management: Reducing mortality in the European Union. Oncologist. 2015;20(5):495–498. 25888268 10.1634/theoncologist.2015-0011 PMC4425394

[r24] European Society for Medical Oncology. Death Rates from Ovarian Cancer will Fall in the EU and UK in 2022. Lugano: ESMO; 2022.

[r25] Liontos M, Papatheodoridi A, Andrikopoulou A, et al. Management of the elderly patients with high‑grade serous ovarian cancer in the real‑world setting. Curr Oncol. 2021;28(2):1143–1152. 33800101 10.3390/curroncol28020110 PMC8025751

[r26] Wentzensen N, Poole EM, Trabert B, et al. Ovarian cancer risk factors by histologic subtype: An analysis from the ovarian cancer cohort consortium. J Clin Oncol. 2016;34(24):2888–2898. 27325851 10.1200/JCO.2016.66.8178 PMC5012665

[r27] European Union. Population Structure and Ageing. Luxembourg: Publications Office of the European Union; 2025.

[r28] Coburn SB, Bray F, Sherman ME, Trabert B. International patterns and trends in ovarian cancer incidence, overall and by histologic subtype. Int J Cancer. 2017;140(11):2451–2460. 28257597 10.1002/ijc.30676 PMC5595147

[r29] Plaxe SC, Braly PS, Freddo JL, McClay E, Kirmani S, Howell SB. Profiles of women age 30‑39 and age less than 30 with epithelial ovarian cancer. Obstet Gynecol. 1993;81(5):651–654. 8469449

[r30] Rodriguez M, Nguyen HN, Averette HE, et al. National survey of ovarian carcinoma XII: Epithelial ovarian malignancies in women less than or equal to 25 years of age. Cancer. 1994;73(4):1245–1250. 8313329 10.1002/1097-0142(19940215)73:4<1245::aid-cncr2820730419>3.0.co;2-5

[r31] Chan JK, Urban R, Cheung MK, et al. Ovarian cancer in younger vs older women: A population‑based analysis. Br J Cancer. 2006;95(10):1314–1320. 17088903 10.1038/sj.bjc.6603457 PMC2360593

[r32] Lim N, Hickey M, Young GP, Macrae FA, Kelly C. Screening and risk reducing surgery for endometrial or ovarian cancers in Lynch syndrome: A systematic review. Int J Gynecol Cancer. 2022;32(5):646–655. 35437274 10.1136/ijgc-2021-003132 PMC9067008

[r33] European Network of Gynaecological Cancer Advocacy Groups. Ovarian Cancer Factsheet. Brussels: ENGAGe; 2018.

[r34] Mazidimoradi A, Momenimovahed Z, Khalajinia Z, Allahqoli L, Salehiniya H, Alkatout I. The global incidence, mortality, and burden of uterine cancer in 2019 and correlation with SDI, tobacco, dietary risks, and metabolic risk factors: An ecological study. Health Sci Rep. 2024;7(1):e1835. 38274134 10.1002/hsr2.1835 PMC10808991

[r35] Colombo N, Creutzberg C, Amant F, et al. ESMO‑ESGO‑ESTRO consensus conference on endometrial cancer: Diagnosis, treatment and follow‑up. Ann Oncol. 2016:27(1):16–41. 26634381 10.1093/annonc/mdv484

[r36] Sung H, Ferlay J, Siegel RL, et al. Global cancer statistics 2020: GLOBOCAN estimates of incidence and mortality worldwide for 36 cancers in 185 countries. CA Cancer J Clin. 2021;71(3):209–249. 33538338 10.3322/caac.21660

[r37] Rauh‑Hain JA, Pepin KJ, Meyer LA, et al. Management for elderly women with advanced‑stage, high‑grade endometrial cancer. Obstet Gynecol. 2015;126(6):1198–1206. 26551187 10.1097/AOG.0000000000001140

[r38] Naghavi M, GBD 2021 Italy Subnational Burden of Disease Collaborators. State of health and inequalities among Italian regions from 2000 to 2021: A systematic analysis based on the Global Burden of Disease Study 2021. Lancet Public Health. 2025;10(4):e309–e320. 40175012 10.1016/S2468-2667(25)00045-3 PMC11962357

[r39] Sant M, Minicozzi P, Allemani C, et al. Regional inequalities in cancer care persist in Italy and can influence survival. Cancer Epidemiol. 2012;36(6):541–547. 22770694 10.1016/j.canep.2012.06.006

[r40] Gravdal BH, Lönnberg S, Skare GB, Sulo G, Bjørge T. Cervical cancer in women under 30 years of age in Norway: A population‑based cohort study. BMC Womens Health. 2021;21(1):110. 33736628 10.1186/s12905-021-01242-3 PMC7977265

[r41] Lau H, Juang C, Chen Y, Twu N, Yen M, Chao K. Aggressive characteristics of cervical cancer in young women in Taiwan. Int J Gynaecol Obstet. 2009;107(3):220–223. 19716131 10.1016/j.ijgo.2009.07.029

[r42] Lancucki L, Fender M, Koukari A, et al. A fall‑off in cervical screening coverage of younger women in developed countries. J Med Screen. 2010;17(2):91–96. 20660438 10.1258/jms.2010.010017

[r43] Wang J, Bai Z, Gao X, Zhang N, Wang Z. The effects of age, period, and cohort on the mortality of cervical cancer in three high‐income countries: Canada, Korea, and Italy. Biomed Res Int. 2021;2021:8829122. 33490279 10.1155/2021/8829122 PMC7803425

[r44] Cappelli MG, Fortunato F, Tafuri S, et al. Cervical cancer prevention: An Italian scenario between organised screening and human papillomaviruses vaccination. Eur J Cancer Care. 2018;27(5):e12905. 10.1111/ecc.12905 PMC6175343 30178893

[r45] Calabrò GE, Riccardi MT, D’Ambrosio F, et al. Cervical cancer elimination in Italy: Current scenario and future endeavors for a value based prevention. Front Public Health. 2022;10:1010237. 36530690 10.3389/fpubh.2022.1010237 PMC9747937

