# Supplementary material for: Mortality of Three Major Gynecological Cancers in the European Region: An Age–Period–Cohort Analysis from 1992 to 2021 and Predictions in a 25‑Year Period
Source: Ann Glob Health. 2025 Jun 10;91(1):30. doi: 10.5334/aogh.4688 (PMC12171803; doi:10.5334/aogh.4688)
Supplement: Supplementary Table 3. — The Lexis diagram of GBD 2021 data used for the APC model of cervical cancer mortality in the European Region. [file agh-91-1-4688-s3.pdf]

Table S3. The Lexis diagram of GBD 2021 data used for the APC model of cervical cancer mortality in the European Region

Period (median)	Age groups																Birth cohort (median)
	15-19	20-24	25-29	30-34	35-39	40-44	45-49	50-54	55-59	60-64	65-69	70-74	75-79	80-84	85-89	90-94	
																√	1900-1904 (1902)
															√	√	1905-1909 (1907)
														√	√	√	1910-1914 (1912)
													√	√	√	√	1915-1919 (1917)
												√	√	√	√	√	1920-1924 (1922)
											√	√	√	√	√	√	1925-1929 (1927)
										√	√	√	√	√	√		1930-1934 (1932)
									√	√	√	√	√	√			1935-1939 (1937)
								√	√	√	√	√	√				1940-1944 (1942)
							√	√	√	√	√	√					1945-1949 (1947)
						√	√	√	√	√	√						1950-1954 (1952)
					√	√	√	√	√	√							1955-1959 (1957)
				√	√	√	√	√	√								1960-1964 (1962)
			√	√	√	√	√	√									1965-1969 (1967)
		√	√	√	√	√											1970-1974 (1972)

1992-1996 (1994)	√	√	√	√	√	√											1974-1979 (1977)
1997-2001 (1999)	√	√	√	√	√												1980-1984 (1982)
2002-2006 (2004)	√	√	√	√													1985-1989 (1987)
2007-2011 (2009)	√	√	√														1990-1994 (1992)
2012-2016 (2014)	√	√															1995-1999 (1997)
2017-2021 (2019)	√																2000-2004 (2002)

√ denotes mortality data of each age group from the corresponding period.

Abbreviation: APC, age-period-cohort.
