# Supplementary material for: Mortality of Three Major Gynecological Cancers in the European Region: An Age–Period–Cohort Analysis from 1992 to 2021 and Predictions in a 25‑Year Period
Source: Ann Glob Health. 2025 Jun 10;91(1):30. doi: 10.5334/aogh.4688 (PMC12171803; doi:10.5334/aogh.4688)
Supplement: Supplementary Table 4. — The global trends in the mortality of the three gynecological cancers (ovarian, uterine, and cervical cancers) from 1992 to 2021. [file agh-91-1-4688-s4.pdf]

Table S4. The global trends in the mortality of the three gynecological cancers (uterine, cervical, and ovarian cancers) from 1992 to 2021.

	Deaths number (n)			All-age mortality			Age-standardized mortality (per 100000)			
	Number in 1992	Number in 2021	Percent change of numbers, 1992-2021, %	Rate in 1992, per 100000	Rate in 2021, per 100000	Percent change of rate, 1992-2021, %	Rate in 1992	Rate in 2021	Percent change of rate, 1992-2021, %	Net drift of mortality, % per year
**Ovarian Cancer**										
Global	105252 (97499 to 113521)	185609 (167962 to 201013)	76.3	3.86 (3.58 to 4.16)	4.72 (4.27 to 5.11)	22.3	4.74 (4.4 to 5.11)	4.06 (3.67 to 4.4)	-14.3	-0.53 (-0.58 to -0.48)
African Region	3361 (2418 to 4233)	10899 (8179 to 12903)	224.3	1.24 (0.89 to 1.56)	1.86 (1.39 to 2.2)	50	2.71 (1.97 to 3.39)	3.79 (2.86 to 4.45)	39.9	1.20 (1.04 to 1.36)
Eastern Mediterranean Region	2578 (1949 to 3287)	9375 (7024 to 11978)	263.7	1.36 (1.02 to 1.73)	2.59 (1.94 to 3.31)	90.4	2.71 (2.05 to 3.54)	3.99 (3.01 to 5.03)	47.2	1.49 (1.32 to 1.66)

	Deaths number (n)			All-age mortality			Age-standardized mortality (per 100000)			
	Number in 1992	Number in 2021	Percent change of numbers, 1992-2021, %	Rate in 1992, per 100000	Rate in 2021, per 100000	Percent change of rate, 1992-2021, %	Rate in 1992	Rate in 2021	Percent change of rate, 1992-2021, %	Net drift of mortality, % per year
European Region	46292 (44089 to 48038)	53324 (48537 to 56723)	15.2	10.37 (9.88 to 10.76)	11.12 (10.13 to 11.83)	7.2	7.33 (7.01 to 7.6)	5.87 (5.41 to 6.21)	-19.9	-0.97 (-1.05 to -0.89)
Region of the Americas	22257 (20830 to 23013)	36170 (33288 to 38173)	62.5	5.93 (5.55 to 6.13)	6.91 (6.36 to 7.3)	16.5	6.3 (5.92 to 6.5)	5.03 (4.66 to 5.29)	-20.2	-0.60 (-0.68 to -0.53)
South-East Asia Region	10570 (8331 to 14264)	35907 (31022 to 43339)	239.7	1.61 (1.27 to 2.17)	3.53 (3.05 to 4.26)	119.3	2.62 (2.05 to 3.52)	3.7 (3.21 to 4.45)	41.2	0.86 (0.76 to 0.97)
Western Pacific Region	19770 (16945 to 23213)	38865 (31223 to 47073)	96.6	2.56 (2.19 to 3.01)	4.11 (3.3 to 4.97)	60.5	3.04 (2.62 to 3.56)	2.67 (2.14 to 3.23)	-12.2	-0.74 (-0.84 to -0.63)
**Uterine cancer**										

	Deaths number (n)			All-age mortality			Age-standardized mortality (per 100000)			
	Number in 1992	Number in 2021	Percent change of numbers, 1992-2021, %	Rate in 1992, per 100000	Rate in 2021, per 100000	Percent change of rate, 1992-2021, %	Rate in 1992	Rate in 2021	Percent change of rate, 1992-2021, %	Net drift of mortality, % per year
Global	56631 (50473 to 60387)	97672 (86516 to 108062)	72.5	2.08 (1.85 to 2.21)	2.48 (2.2 to 2.75)	19.2	2.57 (2.29 to 2.74)	2.11 (1.87 to 2.34)	-17.9	-0.98 (-1.03 to -0.92)
African Region	2112 (1699 to 2582)	5267 (4049 to 6557)	149.4	0.78 (0.63 to 0.95)	0.9 (0.69 to 1.12)	15.4	1.84 (1.47 to 2.28)	2.06 (1.57 to 2.54)	12	0.26 (0.01 to 0.50)
Eastern Mediterranean Region	1522 (1277 to 2000)	4215 (3318 to 5500)	176.9	0.8 (0.67 to 1.05)	1.16 (0.92 to 1.52)	45	1.75 (1.46 to 2.35)	1.98 (1.55 to 2.59)	13.1	0.47 (0.20 to 0.74)
European Region	23144 (21964 to 23951)	32031 (28735 to 34200)	38.4	5.18 (4.92 to 5.36)	6.68 (5.99 to 7.14)	29	3.5 (3.33 to 3.61)	3.31 (3.02 to 3.52)	-5.4	-0.50 (-0.62 to -0.37)

	Deaths number (n)			All-age mortality			Age-standardized mortality (per 100000)			
	Number in 1992	Number in 2021	Percent change of numbers, 1992-2021, %	Rate in 1992, per 100000	Rate in 2021, per 100000	Percent change of rate, 1992-2021, %	Rate in 1992	Rate in 2021	Percent change of rate, 1992-2021, %	Net drift of mortality, % per year
Region of the Americas	10479 (9719 to 10894)	22733 (20712 to 24047)	116.9	2.79 (2.59 to 2.9)	4.35 (3.96 to 4.6)	55.9	2.92 (2.72 to 3.04)	3.06 (2.8 to 3.24)	4.8	0.32 (0.18 to 0.45)
South-East Asia Region	4935 (3750 to 5894)	12260 (10081 to 14954)	148.4	0.75 (0.57 to 0.9)	1.2 (0.99 to 1.47)	60	1.33 (1.02 to 1.58)	1.29 (1.07 to 1.58)	-3	-0.51 (-0.68 to -0.35)
Western Pacific Region	14220 (10723 to 16858)	20570 (16535 to 25886)	44.7	1.84 (1.39 to 2.18)	2.17 (1.75 to 2.74)	17.9	2.21 (1.68 to 2.6)	1.39 (1.11 to 1.75)	-37.1	-1.99 (-2.09 to -1.89)
**Cervical cancer**										
Global	214974 (199452 to 231113)	296667 (272059 to 321906)	38	7.88 (7.31 to 8.47)	7.55 (6.92 to 8.19)	-4.2	9.42 (8.75 to 10.12)	6.62 (6.07 to 7.18)	-29.7	-1.17 (-1.23 to -1.12)

	Deaths number (n)			All-age mortality			Age-standardized mortality (per 100000)			
	Number in 1992	Number in 2021	Percent change of numbers, 1992-2021, %	Rate in 1992, per 100000	Rate in 2021, per 100000	Percent change of rate, 1992-2021, %	Rate in 1992	Rate in 2021	Percent change of rate, 1992-2021, %	Net drift of mortality, % per year
African Region	32115 (27746 to 37903)	58861 (49658 to 68898)	83.3	11.83 (10.22 to 13.96)	10.04 (8.47 to 11.75)	-15.1	23.44 (20.26 to 27.6)	18.5 (15.76 to 21.62)	-21.1	-0.74 (-0.81 to -0.67)
Eastern Mediterranean Region	4981 (4371 to 5723)	9771 (8192 to 11537)	96.2	2.62 (2.3 to 3.01)	2.7 (2.26 to 3.19)	3.1	4.77 (4.2 to 5.5)	3.77 (3.2 to 4.4)	-21	-0.75 (-0.91 to -0.59)
European Region	37986 (36605 to 39058)	30653 (28434 to 32373)	-19.3	8.51 (8.2 to 8.75)	6.39 (5.93 to 6.75)	-24.9	6.31 (6.1 to 6.47)	3.82 (3.59 to 4.04)	-39.5	-1.78 (-1.89 to -1.67)
Region of the Americas	31966 (30756 to 32839)	44259 (40722 to 47551)	38.5	8.52 (8.2 to 8.75)	8.46 (7.78 to 9.09)	-0.7	9.19 (8.86 to 9.44)	6.57 (6.07 to 7.07)	-28.5	-1.19 (-1.25 to -1.13)

	Deaths number (n)			All-age mortality			Age-standardized mortality (per 100000)			
	Number in 1992	Number in 2021	Percent change of numbers, 1992-2021, %	Rate in 1992, per 100000	Rate in 2021, per 100000	Percent change of rate, 1992-2021, %	Rate in 1992	Rate in 2021	Percent change of rate, 1992-2021, %	Net drift of mortality, % per year
South-East Asia Region	64345 (55847 to 73100)	85876 (75505 to 96198)	33.5	9.8 (8.51 to 11.13)	8.44 (7.42 to 9.45)	-13.9	14.92 (12.94 to 17.01)	8.63 (7.59 to 9.66)	-42.2	-1.93 (-2.11 to -1.75)
Western Pacific Region	42049 (36382 to 48912)	66006 (53117 to 81128)	57	5.44 (4.71 to 6.33)	6.98 (5.61 to 8.57)	28.3	6.33 (5.49 to 7.34)	4.63 (3.72 to 5.7)	-26.9	-1.01 (-1.08 to -0.93)
