# Supplementary material for: Mortality of Three Major Gynecological Cancers in the European Region: An Age–Period–Cohort Analysis from 1992 to 2021 and Predictions in a 25‑Year Period
Source: Ann Glob Health. 2025 Jun 10;91(1):30. doi: 10.5334/aogh.4688 (PMC12171803; doi:10.5334/aogh.4688)
Supplement: Supplementary Table 5. — The mortality of ovarian cancer in all countries of the European Region from 1992 to 2021. [file agh-91-1-4688-s5.pdf]

Table S5. The mortality of ovarian cancer in all countries of the European Region from 1992 to 2021

	Deaths number (n)			All-age mortality			Age-standardized mortality (per 100000)			
Location	Number in 1992	Number in 2021	Percent change of numbers, 1992-2021, %	Rate in 1992	Rate in 2021	Percent change of rate, 1992-2021, %	Rate in 1992	Rate in 2021	Percent change of rate, 1992-2021, %	Net drift of mortality, % per year
Albania	24 (19 to 30)	51 (35 to 70)	112.5	1.46 (1.17 to 1.85)	3.82 (2.65 to 5.3)	161.6	2.06 (1.64 to 2.59)	2.29 (1.6 to 3.18)	11.2	0.57 (-1.87 to 3.07)
Andorra	1 (1 to 1)	1 (1 to 2)	0	2.83 (1.84 to 4.15)	3.42 (2.32 to 4.67)	20.8	2.62 (1.71 to 3.84)	1.84 (1.26 to 2.53)	-29.8	-2.81 (-9.93 to 4.86)
Austria	687 (633 to 729)	531 (455 to 584)	-22.7	16.95 (15.61 to 17.99)	11.67 (9.99 to 12.83)	-31.2	9.64 (8.96 to 10.18)	5.22 (4.6 to 5.67)	-45.9	-1.86 (-2.77 to -0.93)
Belarus	396 (356 to 441)	501 (400 to 614)	26.5	7.12 (6.41 to 7.94)	10.04 (8.02 to 12.32)	41	4.92 (4.43 to 5.49)	5.37 (4.22 to 6.67)	9.1	-0.09 (-0.94 to 0.77)

	Deaths number (n)			All-age mortality			Age-standardized mortality (per 100000)			
Location	Number in 1992	Number in 2021	Percent change of numbers, 1992-2021, %	Rate in 1992	Rate in 2021	Percent change of rate, 1992-2021, %	Rate in 1992	Rate in 2021	Percent change of rate, 1992-2021, %	Net drift of mortality, % per year
Belgium	903 (822 to 964)	711 (612 to 802)	-21.3	17.65 (16.06 to 18.83)	12.21 (10.52 to 13.78)	-30.8	10.13 (9.26 to 10.73)	5.48 (4.85 to 6.08)	-45.9	-2.15 (-2.77 to -1.52)
Bosnia and Herzegovina	120 (97 to 148)	194 (136 to 251)	61.7	5.32 (4.3 to 6.59)	11.47 (8.05 to 14.84)	115.6	4.81 (3.9 to 5.95)	5.81 (4.04 to 7.55)	20.8	0.82 (-0.81 to 2.47)
Bulgaria	373 (344 to 405)	582 (491 to 683)	56	8.59 (7.92 to 9.32)	16.64 (14.03 to 19.51)	93.7	5.77 (5.3 to 6.27)	8.06 (6.7 to 9.62)	39.7	0.97 (-0.004 to 1.95)
Croatia	267 (239 to 289)	335 (282 to 391)	25.5	10.77 (9.64 to 11.67)	15.45 (13.02 to 18.05)	43.5	7.42 (6.6 to 8.05)	6.85 (5.82 to 7.9)	-7.7	-0.02 (-1.29 to 1.28)
Cyprus	31 (24 to 44)	60 (43 to 79)	93.5	7.82 (5.95 to 10.98)	8.75 (6.19 to 11.45)	11.9	7.21 (5.52 to 10.19)	5.58 (4.06 to 7.28)	-22.6	-0.82 (-3.35 to 1.77)

	Deaths number (n)			All-age mortality			Age-standardized mortality (per 100000)			
Location	Number in 1992	Number in 2021	Percent change of numbers, 1992-2021, %	Rate in 1992	Rate in 2021	Percent change of rate, 1992-2021, %	Rate in 1992	Rate in 2021	Percent change of rate, 1992-2021, %	Net drift of mortality, % per year
Czechia	572 (530 to 624)	764 (649 to 888)	33.6	10.8 (10.01 to 11.77)	14.14 (12.02 to 16.44)	30.9	7.29 (6.78 to 7.93)	6.66 (5.67 to 7.68)	-8.6	-0.35 (-0.96 to 0.27)
Denmark	369 (327 to 408)	431 (376 to 476)	16.8	14.07 (12.47 to 15.55)	14.67 (12.8 to 16.21)	4.3	8.62 (7.69 to 9.48)	6.66 (5.93 to 7.3)	-22.7	-2.02 (-3.10 to -0.93)
Estonia	95 (83 to 106)	93 (76 to 111)	-2.1	11.5 (10.09 to 12.87)	13.4 (10.96 to 16.04)	16.5	7.52 (6.63 to 8.41)	5.95 (4.96 to 7.06)	-20.9	-1.23 (-3.16 to 0.74)
Finland	359 (332 to 383)	369 (316 to 414)	2.8	13.8 (12.74 to 14.71)	13.15 (11.28 to 14.78)	-4.7	8.34 (7.75 to 8.86)	5.32 (4.66 to 5.86)	-36.2	-1.28 (-2.40 to -0.15)
France	3715 (3378 to 3975)	4356 (3697 to 4950)	17.3	12.46 (11.33 to 13.33)	12.74 (10.81 to 14.47)	2.2	7.73 (7.12 to 8.23)	5.38 (4.7 to 6.03)	-30.4	-1.39 (-1.68 to -1.11)

	Deaths number (n)			All-age mortality			Age-standardized mortality (per 100000)			
Location	Number in 1992	Number in 2021	Percent change of numbers, 1992-2021, %	Rate in 1992	Rate in 2021	Percent change of rate, 1992-2021, %	Rate in 1992	Rate in 2021	Percent change of rate, 1992-2021, %	Net drift of mortality, % per year
Germany	7404 (6719 to 8007)	6009 (5132 to 6708)	-18.8	17.79 (16.14 to 19.23)	14.03 (11.98 to 15.66)	-21.1	9.61 (8.79 to 10.31)	5.75 (5.06 to 6.3)	-40.2	-1.8 (-2.04 to -1.56)
Greece	551 (510 to 586)	767 (673 to 836)	39.2	10.32 (9.55 to 10.96)	14.65 (12.87 to 15.98)	42	6.59 (6.12 to 6.98)	6.12 (5.52 to 6.58)	-7.1	-0.74 (-1.55 to 0.08)
Hungary	677 (609 to 742)	716 (618 to 810)	5.8	12.55 (11.29 to 13.75)	14.26 (12.31 to 16.13)	13.6	8.09 (7.28 to 8.88)	6.66 (5.74 to 7.53)	-17.7	-0.71 (-1.35 to -0.07)
Iceland	14 (13 to 15)	17 (14 to 19)	21.4	10.8 (9.78 to 11.79)	9.83 (8.24 to 11.04)	-9	8.99 (8.18 to 9.79)	5.64 (4.82 to 6.31)	-37.3	-1.84 (-5.54 to 2.01)
Ireland	246 (230 to 263)	279 (242 to 312)	13.4	13.58 (12.66 to 14.49)	11.15 (9.7 to 12.51)	-17.9	11.15 (10.38 to 11.86)	6.72 (5.91 to 7.49)	-39.7	-1.92 (-3.17 to -0.65)

	Deaths number (n)			All-age mortality			Age-standardized mortality (per 100000)			
Location	Number in 1992	Number in 2021	Percent change of numbers, 1992-2021, %	Rate in 1992	Rate in 2021	Percent change of rate, 1992-2021, %	Rate in 1992	Rate in 2021	Percent change of rate, 1992-2021, %	Net drift of mortality, % per year
Israel	234 (217 to 250)	335 (288 to 370)	43.2	8.84 (8.19 to 9.43)	6.96 (5.97 to 7.69)	-21.3	8.45 (7.82 to 9)	5.02 (4.39 to 5.51)	-40.6	-2.11 (-3.20 to -1.02)
Italy	3129 (2902 to 3295)	4134 (3506 to 4538)	32.1	10.71 (9.94 to 11.28)	13.47 (11.42 to 14.79)	25.8	6.21 (5.84 to 6.49)	5.31 (4.71 to 5.72)	-14.5	-0.60 (-0.89 to -0.30)
Latvia	141 (126 to 157)	184 (158 to 213)	30.5	9.98 (8.91 to 11.1)	18.24 (15.62 to 21.1)	82.8	6.48 (5.78 to 7.21)	8.37 (7.09 to 9.73)	29.2	0.85 (-0.78 to 2.49)
Lithuania	229 (204 to 249)	250 (209 to 287)	9.2	11.77 (10.49 to 12.8)	17 (14.17 to 19.52)	44.4	8.34 (7.46 to 9.07)	7.56 (6.33 to 8.74)	-9.4	-1.30 (-2.53 to -0.06)
Luxembourg	34 (32 to 37)	37 (33 to 41)	8.8	17.42 (16.19 to 18.74)	11.63 (10.26 to 12.9)	-33.2	10.76 (10.03 to 11.55)	6.52 (5.83 to 7.25)	-39.4	-1.66 (-4.48 to 1.25)

	Deaths number (n)			All-age mortality			Age-standardized mortality (per 100000)			
Location	Number in 1992	Number in 2021	Percent change of numbers, 1992-2021, %	Rate in 1992	Rate in 2021	Percent change of rate, 1992-2021, %	Rate in 1992	Rate in 2021	Percent change of rate, 1992-2021, %	Net drift of mortality, % per year
Malta	21 (19 to 23)	35 (30 to 40)	66.7	11.13 (10.09 to 12.13)	15.88 (13.7 to 18.01)	42.7	8.6 (7.83 to 9.36)	6.98 (6.11 to 7.88)	-18.8	-0.1 (-3.14 to 3.04)
Monaco	2 (2 to 4)	3 (2 to 5)	50	15.27 (9.43 to 25.35)	15.07 (9.86 to 25.3)	-1.3	6.67 (4.11 to 10.87)	5.84 (3.81 to 9.75)	-12.4	-0.71 (-7.98 to 7.13)
Montenegro	18 (14 to 24)	29 (21 to 38)	61.1	5.8 (4.3 to 7.52)	9.22 (6.63 to 12.26)	59	5.06 (3.75 to 6.57)	5.48 (3.94 to 7.27)	8.3	0.36 (-2.64 to 3.46)
Netherlands	1093 (1001 to 1161)	1161 (1012 to 1293)	6.2	14.28 (13.08 to 15.17)	13.4 (11.68 to 14.92)	-6.2	9.49 (8.76 to 10.05)	6.14 (5.42 to 6.78)	-35.3	-1.84 (-2.36 to -1.33)
North Macedonia	63 (53 to 79)	120 (88 to 158)	90.5	6.34 (5.3 to 7.92)	11.15 (8.21 to 14.71)	75.9	6.09 (5.1 to 7.61)	7.05 (5.23 to 9.24)	15.8	0.97 (-1.04 to 3.01)

	Deaths number (n)			All-age mortality			Age-standardized mortality (per 100000)			
Location	Number in 1992	Number in 2021	Percent change of numbers, 1992-2021, %	Rate in 1992	Rate in 2021	Percent change of rate, 1992-2021, %	Rate in 1992	Rate in 2021	Percent change of rate, 1992-2021, %	Net drift of mortality, % per year
Norway	331 (307 to 347)	347 (306 to 375)	4.8	15.25 (14.15 to 15.99)	12.94 (11.4 to 14)	-15.1	9.14 (8.63 to 9.53)	6.42 (5.79 to 6.88)	-29.8	-1.74 (-3.01 to -0.44)
Poland	2276 (2196 to 2346)	3505 (3117 to 3871)	54	11.56 (11.15 to 11.92)	17.76 (15.8 to 19.62)	53.6	9.1 (8.79 to 9.37)	8.81 (7.84 to 9.76)	-3.2	-0.30 (-0.60 to 0.002)
Portugal	356 (331 to 382)	479 (412 to 532)	34.6	6.79 (6.3 to 7.27)	8.6 (7.4 to 9.56)	26.7	4.52 (4.21 to 4.82)	3.61 (3.21 to 3.97)	-20.1	-1.54 (-2.44 to -0.62)
Republic of Moldova	152 (141 to 161)	134 (116 to 151)	-11.8	6.54 (6.09 to 6.93)	7.09 (6.17 to 8.02)	8.4	5.69 (5.3 to 6.03)	3.99 (3.48 to 4.51)	-29.9	-0.72 (-2.58 to 1.16)
Romania	838 (783 to 890)	1329 (1156 to 1509)	58.6	7.08 (6.62 to 7.53)	13.66 (11.88 to 15.51)	92.9	5.51 (5.16 to 5.83)	6.78 (5.87 to 7.69)	23	0.40 (-0.12 to 0.92)

	Deaths number (n)			All-age mortality			Age-standardized mortality (per 100000)			
Location	Number in 1992	Number in 2021	Percent change of numbers, 1992-2021, %	Rate in 1992	Rate in 2021	Percent change of rate, 1992-2021, %	Rate in 1992	Rate in 2021	Percent change of rate, 1992-2021, %	Net drift of mortality, % per year
Russian Federation	7914 (7611 to 8179)	8707 (7746 to 9617)	10	9.83 (9.45 to 10.15)	11.25 (10.01 to 12.42)	14.4	6.96 (6.69 to 7.19)	6.21 (5.53 to 6.86)	-10.8	-0.29 (-0.47 to -0.11)
San Marino	1 (1 to 1)	1 (1 to 1)	0	7.45 (5.7 to 9.84)	5.57 (3.42 to 8.09)	-25.2	4.52 (3.47 to 5.98)	2.46 (1.47 to 3.59)	-45.6	-1.98 (-9.15 to 5.76)
Serbia	408 (316 to 523)	620 (432 to 779)	52	8.28 (6.41 to 10.62)	13.87 (9.67 to 17.43)	67.5	6.6 (5.09 to 8.46)	7.22 (5.14 to 8.95)	9.4	0.18 (-0.58 to 0.91)
Slovakia	257 (208 to 297)	359 (259 to 456)	39.7	9.41 (7.63 to 10.89)	12.92 (9.32 to 16.4)	37.3	7.52 (6.09 to 8.69)	6.89 (4.98 to 8.7)	-8.4	-0.55 (-1.71 to 0.61)
Slovenia	107 (98 to 115)	129 (108 to 151)	20.6	10.47 (9.57 to 11.3)	12.35 (10.38 to 14.46)	18	7.25 (6.62 to 7.82)	5.41 (4.55 to 6.36)	-25.4	-0.55 (-2.46 to 1.41)

	Deaths number (n)			All-age mortality			Age-standardized mortality (per 100000)			
Location	Number in 1992	Number in 2021	Percent change of numbers, 1992-2021, %	Rate in 1992	Rate in 2021	Percent change of rate, 1992-2021, %	Rate in 1992	Rate in 2021	Percent change of rate, 1992-2021, %	Net drift of mortality, % per year
Spain	1668 (1506 to 1794)	2296 (1941 to 2654)	37.6	8.38 (7.57 to 9.02)	9.86 (8.33 to 11.39)	17.7	5.48 (4.99 to 5.87)	4.43 (3.83 to 5.01)	-19.2	-1.12 (-1.50 to -0.74)
Sweden	732 (671 to 776)	641 (537 to 736)	-12.4	16.63 (15.24 to 17.61)	12.44 (10.41 to 14.27)	-25.2	9.28 (8.69 to 9.75)	5.51 (4.73 to 6.31)	-40.6	-1.91 (-2.70 to -1.10)
Switzerland	305 (263 to 341)	446 (372 to 513)	46.2	8.61 (7.43 to 9.65)	9.97 (8.33 to 11.48)	15.8	5.17 (4.49 to 5.73)	4.41 (3.78 to 5.04)	-14.7	-1.92 (-3.08 to -0.74)
Ukraine	2265 (2015 to 2520)	2316 (1495 to 3318)	2.3	8.05 (7.16 to 8.96)	10.02 (6.46 to 14.35)	24.5	5.27 (4.7 to 5.88)	5.4 (3.42 to 7.82)	2.5	-0.05 (-0.40 to 0.30)

	Deaths number (n)			All-age mortality			Age-standardized mortality (per 100000)			
Location	Number in 1992	Number in 2021	Percent change of numbers, 1992-2021, %	Rate in 1992	Rate in 2021	Percent change of rate, 1992-2021, %	Rate in 1992	Rate in 2021	Percent change of rate, 1992-2021, %	Net drift of mortality, % per year
United Kingdom	5104 (4854 to 5241)	4765 (4276 to 5021)	-6.6	17.22 (16.38 to 17.68)	13.79 (12.38 to 14.53)	-19.9	10.43 (10.04 to 10.67)	6.79 (6.22 to 7.1)	-34.9	-1.57 (-1.82 to -1.32)
