# Supplementary material for: Mortality of Three Major Gynecological Cancers in the European Region: An Age–Period–Cohort Analysis from 1992 to 2021 and Predictions in a 25‑Year Period
Source: Ann Glob Health. 2025 Jun 10;91(1):30. doi: 10.5334/aogh.4688 (PMC12171803; doi:10.5334/aogh.4688)
Supplement: Supplementary Table 6. — The mortality of uterine cancer in all countries of the European Region from 1992 to 2021. [file agh-91-1-4688-s6.pdf]

Table S6. The mortality of uterine cancer in all countries of the European Region from 1992 to 2021

	Deaths number (n)			All-age mortality			Age-standardized mortality (per 100000)			
Location	Number in 1992	Number in 2021	Percent change of numbers, 1992-2021, %	Rate in 1992, per 100000	Rate in 2021, per 100000	Percent change of rate, 1992-2021, %	Rate in 1992	Rate in 2021	Percent change of rate, 1992-2021, %	Net drift of mortality, % per year
Albania	25 (17 to 35)	45 (29 to 66)	80	1.51 (1.07 to 2.13)	3.38 (2.18 to 4.95)	123.8	2.23 (1.56 to 3.16)	1.94 (1.27 to 2.81)	-13	-0.32 (-3.12 to 2.56)
Andorra	1 (0 to 1)	1 (1 to 2)	0	2.06 (1.37 to 3.02)	2.76 (1.79 to 3.97)	34	1.92 (1.28 to 2.85)	1.42 (0.91 to 2.04)	-26	-2.75 (-10.06 to 5.15)
Austria	268 (241 to 294)	262 (218 to 295)	-2.2	6.61 (5.95 to 7.26)	5.75 (4.8 to 6.48)	-13	3.46 (3.13 to 3.77)	2.38 (2.05 to 2.67)	-31.2	-1.00 (-2.86 to 0.89)
Belarus	284 (256 to 319)	413 (329 to 500)	45.4	5.11 (4.6 to 5.75)	8.28 (6.6 to 10.04)	62	3.34 (3.01 to 3.74)	4.01 (3.19 to 4.86)	20.1	-0.48 (-1.88 to 0.94)

	Deaths number (n)			All-age mortality			Age-standardized mortality (per 100000)			
Location	Number in 1992	Number in 2021	Percent change of numbers, 1992-2021, %	Rate in 1992, per 100000	Rate in 2021, per 100000	Percent change of rate, 1992-2021, %	Rate in 1992	Rate in 2021	Percent change of rate, 1992-2021, %	Net drift of mortality, % per year
Belgium	299 (266 to 328)	366 (310 to 409)	22.4	5.85 (5.2 to 6.4)	6.28 (5.33 to 7.02)	7.4	3.12 (2.8 to 3.4)	2.6 (2.25 to 2.89)	-16.7	-0.69 (-2.48 to 1.14)
Bosnia and Herzegovina	64 (48 to 87)	95 (65 to 128)	48.4	2.85 (2.14 to 3.85)	5.61 (3.83 to 7.57)	96.8	2.66 (2 to 3.54)	2.67 (1.81 to 3.62)	0.4	-0.02 (-2.43 to 2.45)
Bulgaria	323 (261 to 390)	482 (384 to 585)	49.2	7.45 (6.01 to 8.99)	13.76 (10.98 to 16.72)	84.7	4.78 (3.9 to 5.74)	5.95 (4.74 to 7.27)	24.5	1.85 (0.23 to 3.49)
Croatia	138 (122 to 156)	181 (153 to 211)	31.2	5.59 (4.93 to 6.31)	8.35 (7.07 to 9.75)	49.4	3.77 (3.33 to 4.25)	3.36 (2.88 to 3.89)	-10.9	0.41 (-1.62 to 2.49)

	Deaths number (n)			All-age mortality			Age-standardized mortality (per 100000)			
Location	Number in 1992	Number in 2021	Percent change of numbers, 1992-2021, %	Rate in 1992, per 100000	Rate in 2021, per 100000	Percent change of rate, 1992-2021, %	Rate in 1992	Rate in 2021	Percent change of rate, 1992-2021, %	Net drift of mortality, % per year
Cyprus	18 (12 to 27)	30 (21 to 38)	66.7	4.43 (3.06 to 6.65)	4.32 (3.08 to 5.54)	-2.5	4.28 (2.92 to 6.45)	2.74 (1.96 to 3.52)	-36	-1.92 (-5.80 to 2.12)
Czechia	449 (379 to 534)	468 (376 to 582)	4.2	8.47 (7.15 to 10.08)	8.67 (6.97 to 10.79)	2.4	5.33 (4.5 to 6.33)	3.71 (2.96 to 4.63)	-30.4	-1.02 (-2.38 to 0.36)
Denmark	183 (163 to 198)	174 (151 to 194)	-4.9	6.98 (6.21 to 7.56)	5.92 (5.13 to 6.6)	-15.2	3.81 (3.41 to 4.14)	2.56 (2.23 to 2.85)	-32.8	-1.70 (-3.96 to 0.62)
Estonia	50 (45 to 57)	50 (41 to 59)	0	6.12 (5.43 to 6.96)	7.26 (5.93 to 8.54)	18.6	3.77 (3.32 to 4.31)	2.91 (2.35 to 3.44)	-22.8	-0.98 (-3.98 to 2.13)
Finland	162 (145 to 178)	242 (205 to 273)	49.4	6.23 (5.57 to 6.85)	8.64 (7.3 to 9.76)	38.7	3.49 (3.14 to 3.82)	3.14 (2.7 to 3.51)	-10	-0.81 (-3.09 to 1.52)

	Deaths number (n)			All-age mortality			Age-standardized mortality (per 100000)			
Location	Number in 1992	Number in 2021	Percent change of numbers, 1992-2021, %	Rate in 1992, per 100000	Rate in 2021, per 100000	Percent change of rate, 1992-2021, %	Rate in 1992	Rate in 2021	Percent change of rate, 1992-2021, %	Net drift of mortality, % per year
France	1594 (1403 to 1769)	2281 (1907 to 2560)	43.1	5.35 (4.71 to 5.93)	6.67 (5.57 to 7.48)	24.7	3.02 (2.7 to 3.34)	2.59 (2.22 to 2.88)	-14.2	-0.65 (-1.46 to 0.17)
Germany	2545 (2269 to 2788)	2406 (2058 to 2725)	-5.5	6.11 (5.45 to 6.7)	5.61 (4.8 to 6.36)	-8.2	3.02 (2.72 to 3.29)	2.11 (1.85 to 2.35)	-30.1	-1.03 (-1.87 to -0.18)
Greece	215 (200 to 228)	394 (346 to 426)	83.3	4.03 (3.74 to 4.27)	7.53 (6.61 to 8.15)	86.8	2.47 (2.3 to 2.61)	2.9 (2.61 to 3.1)	17.4	1.00 (-0.87 to 2.89)
Hungary	465 (404 to 526)	427 (359 to 514)	-8.2	8.62 (7.49 to 9.74)	8.5 (7.15 to 10.23)	-1.4	5.23 (4.54 to 5.9)	3.65 (3.04 to 4.4)	-30.2	-1.83 (-3.12 to -0.53)
Iceland	4 (4 to 4)	8 (6 to 9)	100	3.08 (2.72 to 3.38)	4.38 (3.65 to 5.03)	42.2	2.42 (2.15 to 2.67)	2.34 (1.99 to 2.67)	-3.3	-0.7 (-6.23 to 5.16)

	Deaths number (n)			All-age mortality			Age-standardized mortality (per 100000)			
Location	Number in 1992	Number in 2021	Percent change of numbers, 1992-2021, %	Rate in 1992, per 100000	Rate in 2021, per 100000	Percent change of rate, 1992-2021, %	Rate in 1992	Rate in 2021	Percent change of rate, 1992-2021, %	Net drift of mortality, % per year
Ireland	68 (62 to 75)	108 (91 to 123)	58.8	3.77 (3.41 to 4.14)	4.34 (3.66 to 4.92)	15.1	2.87 (2.6 to 3.13)	2.5 (2.13 to 2.83)	-12.9	0.30 (-2.26 to 2.92)
Israel	72 (64 to 79)	177 (149 to 199)	145.8	2.7 (2.42 to 2.98)	3.67 (3.09 to 4.14)	35.9	2.5 (2.25 to 2.76)	2.47 (2.08 to 2.79)	-1.2	-0.002 (-2.28 to 2.33)
Italy	589 (535 to 627)	1933 (1624 to 2126)	228.2	2.02 (1.83 to 2.15)	6.3 (5.29 to 6.93)	211.9	1.08 (0.99 to 1.14)	2.29 (1.99 to 2.48)	112	2.89 (1.97 to 3.82)
Latvia	87 (78 to 97)	122 (103 to 140)	40.2	6.17 (5.5 to 6.87)	12.06 (10.19 to 13.89)	95.5	3.75 (3.35 to 4.17)	4.67 (3.92 to 5.38)	24.5	0.46 (-1.95 to 2.93)
Lithuania	104 (93 to 117)	153 (129 to 177)	47.1	5.32 (4.76 to 5.98)	10.37 (8.77 to 12.04)	94.9	3.63 (3.24 to 4.08)	4.22 (3.51 to 4.93)	16.3	0.69 (-1.34 to 2.76)

	Deaths number (n)			All-age mortality			Age-standardized mortality (per 100000)			
Location	Number in 1992	Number in 2021	Percent change of numbers, 1992-2021, %	Rate in 1992, per 100000	Rate in 2021, per 100000	Percent change of rate, 1992-2021, %	Rate in 1992	Rate in 2021	Percent change of rate, 1992-2021, %	Net drift of mortality, % per year
Luxembourg	16 (14 to 17)	20 (17 to 22)	25	7.86 (7.27 to 8.51)	6.22 (5.41 to 6.93)	-20.9	4.56 (4.24 to 4.93)	3.27 (2.87 to 3.63)	-28.3	-0.54 (-4.69 to 3.80)
Malta	9 (8 to 10)	18 (15 to 21)	100	4.55 (4.03 to 5.09)	8.19 (6.94 to 9.59)	80	3.44 (3.05 to 3.85)	3.23 (2.77 to 3.78)	-6.1	-0.62 (-5.27 to 4.25)
Monaco	1 (0 to 1)	1 (1 to 1)	0	3.51 (2.2 to 5.22)	4.37 (2.85 to 5.99)	24.5	1.29 (0.82 to 1.92)	1.5 (0.99 to 2.05)	16.3	-0.67 (-8.13 to 7.40)
Montenegro	10 (7 to 14)	18 (13 to 25)	80	3.14 (2.22 to 4.49)	5.64 (4.03 to 7.84)	79.6	2.73 (1.93 to 3.9)	3.22 (2.31 to 4.47)	17.9	0.05 (-4.06 to 4.34)
Netherlands	322 (288 to 355)	479 (398 to 542)	48.8	4.21 (3.76 to 4.63)	5.53 (4.59 to 6.26)	31.4	2.56 (2.31 to 2.8)	2.37 (2 to 2.67)	-7.4	-0.61 (-2.05 to 0.86)

	Deaths number (n)			All-age mortality			Age-standardized mortality (per 100000)			
Location	Number in 1992	Number in 2021	Percent change of numbers, 1992-2021, %	Rate in 1992, per 100000	Rate in 2021, per 100000	Percent change of rate, 1992-2021, %	Rate in 1992	Rate in 2021	Percent change of rate, 1992-2021, %	Net drift of mortality, % per year
North Macedonia	45 (36 to 59)	78 (59 to 105)	73.3	4.54 (3.64 to 5.99)	7.31 (5.49 to 9.82)	61	4.54 (3.64 to 6.02)	4.63 (3.46 to 6.18)	2	-0.23 (-2.69 to 2.30)
Norway	124 (112 to 131)	135 (116 to 148)	8.9	5.73 (5.18 to 6.05)	5.05 (4.34 to 5.51)	-11.9	3.04 (2.81 to 3.21)	2.29 (2.02 to 2.48)	-24.7	-1.26 (-3.64 to 1.17)
Poland	1077 (1021 to 1130)	2278 (2014 to 2519)	111.5	5.47 (5.19 to 5.74)	11.55 (10.21 to 12.77)	111.2	4.06 (3.86 to 4.26)	5.08 (4.52 to 5.63)	25.1	0.12 (-0.74 to 0.98)
Portugal	293 (259 to 327)	403 (331 to 465)	37.5	5.58 (4.92 to 6.23)	7.23 (5.95 to 8.35)	29.6	3.53 (3.14 to 3.93)	2.65 (2.23 to 3.05)	-24.9	-1.08 (-2.83 to 0.71)
Republic of Moldova	94 (87 to 101)	112 (101 to 127)	19.1	4.04 (3.77 to 4.35)	5.97 (5.37 to 6.72)	47.8	3.46 (3.24 to 3.72)	3.15 (2.84 to 3.55)	-9	-1.12 (-3.14 to 0.95)

	Deaths number (n)			All-age mortality			Age-standardized mortality (per 100000)			
Location	Number in 1992	Number in 2021	Percent change of numbers, 1992-2021, %	Rate in 1992, per 100000	Rate in 2021, per 100000	Percent change of rate, 1992-2021, %	Rate in 1992	Rate in 2021	Percent change of rate, 1992-2021, %	Net drift of mortality, % per year
Romania	533 (470 to 605)	676 (574 to 787)	26.8	4.51 (3.97 to 5.11)	6.94 (5.9 to 8.09)	53.9	3.38 (3 to 3.8)	3.2 (2.69 to 3.75)	-5.3	-0.40 (-1.47 to 0.68)
Russian Federation	5828 (5582 to 6045)	7356 (6567 to 8042)	26.2	7.23 (6.93 to 7.5)	9.5 (8.48 to 10.39)	31.4	4.87 (4.66 to 5.05)	4.85 (4.32 to 5.33)	-0.4	-0.89 (-1.13 to -0.65)
San Marino	0 (0 to 0)	0 (0 to 0)	0	1.43 (1.06 to 2.01)	1.34 (0.82 to 2.03)	-6.3	0.81 (0.59 to 1.14)	0.53 (0.31 to 0.81)	-34.6	-1.98 (-9.34 to 5.98)
Serbia	250 (170 to 350)	343 (237 to 481)	37.2	5.07 (3.44 to 7.11)	7.67 (5.31 to 10.77)	51.3	4.14 (2.79 to 5.81)	3.67 (2.54 to 5.17)	-11.4	-0.48 (-1.92 to 0.99)
Slovakia	215 (161 to 275)	262 (178 to 359)	21.9	7.89 (5.91 to 10.07)	9.43 (6.41 to 12.93)	19.5	6.02 (4.52 to 7.67)	4.66 (3.19 to 6.42)	-22.6	-0.86 (-2.32 to 0.61)

	Deaths number (n)			All-age mortality			Age-standardized mortality (per 100000)			
Location	Number in 1992	Number in 2021	Percent change of numbers, 1992-2021, %	Rate in 1992, per 100000	Rate in 2021, per 100000	Percent change of rate, 1992-2021, %	Rate in 1992	Rate in 2021	Percent change of rate, 1992-2021, %	Net drift of mortality, % per year
Slovenia	63 (57 to 71)	88 (71 to 106)	39.7	6.23 (5.57 to 6.96)	8.47 (6.83 to 10.13)	36	4.07 (3.65 to 4.56)	3.33 (2.68 to 4.06)	-18.2	-0.62 (-3.17 to 2.00)
Spain	1052 (944 to 1156)	1501 (1245 to 1723)	42.7	5.29 (4.74 to 5.81)	6.45 (5.35 to 7.4)	21.9	3.16 (2.86 to 3.45)	2.55 (2.16 to 2.88)	-19.3	-0.54 (-1.46 to 0.40)
Sweden	266 (235 to 293)	331 (271 to 388)	24.4	6.03 (5.32 to 6.65)	6.42 (5.25 to 7.53)	6.5	2.85 (2.56 to 3.13)	2.53 (2.1 to 2.95)	-11.2	-0.97 (-3.27 to 1.39)
Switzerland	181 (157 to 202)	226 (184 to 263)	24.9	5.11 (4.45 to 5.71)	5.06 (4.11 to 5.89)	-1	2.75 (2.44 to 3.03)	2.1 (1.78 to 2.42)	-23.6	-0.62 (-2.56 to 1.36)
Ukraine	1926 (1743 to 2121)	2078 (1417 to 2928)	7.9	6.84 (6.2 to 7.54)	8.99 (6.13 to 12.66)	31.4	4.09 (3.74 to 4.49)	4.38 (2.96 to 6.23)	7.1	-0.16 (-0.62 to 0.3)

	Deaths number (n)			All-age mortality			Age-standardized mortality (per 100000)			
Location	Number in 1992	Number in 2021	Percent change of numbers, 1992-2021, %	Rate in 1992, per 100000	Rate in 2021, per 100000	Percent change of rate, 1992-2021, %	Rate in 1992	Rate in 2021	Percent change of rate, 1992-2021, %	Net drift of mortality, % per year
United Kingdom	1336 (1253 to 1382)	2524 (2235 to 2678)	88.9	4.51 (4.23 to 4.66)	7.31 (6.47 to 7.75)	62.1	2.42 (2.29 to 2.49)	3.36 (3.03 to 3.54)	38.8	1.91 (1.01 to 2.82)
