# Supplementary material for: Mortality of Three Major Gynecological Cancers in the European Region: An Age–Period–Cohort Analysis from 1992 to 2021 and Predictions in a 25‑Year Period
Source: Ann Glob Health. 2025 Jun 10;91(1):30. doi: 10.5334/aogh.4688 (PMC12171803; doi:10.5334/aogh.4688)
Supplement: Supplementary Table 7. — The mortality of cervical cancer in all countries of the European Region from 1992 to 2021. [file agh-91-1-4688-s7.pdf]

Table S7. The mortality of cervical cancer in all countries of the European Region from 1992 to 2021.

	Deaths number (n)			All-age mortality			Age-standardized mortality (per 100000)			
Location	Number in 1992	Number in 2021	Percent change of numbers, 1992-2021, %	Rate in 1992, per 100000	Rate in 2021, per 100000	Percent change of rate, 1992-2021, %	Rate in 1992	Rate in 2021	Percent change of numbers, 1992-2021, %	Net drift of mortality, % per year
Albania	45 (33 to 61)	54 (37 to 75)	20	2.77 (2.05 to 3.73)	4.03 (2.78 to 5.64)	45.5	3.72 (2.79 to 4.97)	2.59 (1.76 to 3.65)	-30.4	-0.77 (-2.78 to 1.28)

	Deaths number (n)			All-age mortality			Age-standardized mortality (per 100000)			
Location	Number in 1992	Number in 2021	Percent change of numbers, 1992-2021, %	Rate in 1992, per 100000	Rate in 2021, per 100000	Percent change of rate, 1992-2021, %	Rate in 1992	Rate in 2021	Percent change of numbers, 1992-2021, %	Net drift of mortality, % per year
Andorra	1 (0 to 1)	1 (1 to 1)	0	2.39 (1.62 to 3.47)	2.26 (1.58 to 3.22)	-5.4	2.19 (1.49 to 3.16)	1.25 (0.86 to 1.81)	-42.9	-2.81 (-9.93 to 4.86)
Austria	380 (347 to 406)	205 (179 to 227)	-46.1	9.37 (8.56 to 10.03)	4.51 (3.93 to 4.98)	-51.9	5.75 (5.34 to 6.12)	2.23 (1.97 to 2.44)	-61.2	-2.94 (-3.91 to -1.96)
Belarus	664 (622 to 710)	389 (315 to 472)	-41.4	11.95 (11.18 to 12.78)	7.8 (6.31 to 9.47)	-34.7	8.69 (8.14 to 9.28)	4.66 (3.74 to 5.73)	-46.4	-2.33 (-3.19 to -1.47)
Belgium	376 (342 to 406)	273 (235 to 303)	-27.4	7.35 (6.69 to 7.94)	4.69 (4.04 to 5.21)	-36.2	4.52 (4.14 to 4.85)	2.39 (2.14 to 2.65)	-47.1	-2.17 (-3.11 to -1.22)
Bosnia and	171 (139 to 210)	143 (108 to 183)	-16.4	7.6 (6.17 to 9.31)	8.49 (6.38 to 10.85)	11.7	6.89 (5.63 to 8.42)	4.68 (3.46 to 6.02)	-32.1	-0.77 (-2.17 to 0.65)

	Deaths number (n)			All-age mortality			Age-standardized mortality (per 100000)			
Location	Number in 1992	Number in 2021	Percent change of numbers, 1992-2021, %	Rate in 1992, per 100000	Rate in 2021, per 100000	Percent change of rate, 1992-2021, %	Rate in 1992	Rate in 2021	Percent change of numbers, 1992-2021, %	Net drift of mortality, % per year
Herzegovina										
Bulgaria	514 (459 to 576)	504 (416 to 595)	-1.9	11.83 (10.58 to 13.26)	14.41 (11.89 to 17)	21.8	8.5 (7.68 to 9.5)	7.8 (6.43 to 9.17)	-8.2	0.11 (-1.19 to 1.43)
Croatia	241 (208 to 277)	158 (125 to 194)	-34.4	9.73 (8.41 to 11.19)	7.27 (5.77 to 8.95)	-25.3	6.86 (5.93 to 7.87)	3.51 (2.74 to 4.35)	-48.8	-1.46 (-2.76 to -0.15)
Cyprus	19 (16 to 23)	24 (19 to 29)	26.3	4.82 (3.98 to 5.8)	3.45 (2.75 to 4.25)	-28.4	4.69 (3.84 to 5.59)	2.31 (1.84 to 2.83)	-50.7	-2.79 (-5.34 to -0.18)
Czechia	637 (573 to 702)	392 (326 to 470)	-38.5	12.03 (10.82 to 13.26)	7.26 (6.03 to 8.71)	-39.7	8.47 (7.69 to 9.25)	3.7 (3.11 to 4.4)	-56.3	-2.62 (-3.51 to -1.73)

	Deaths number (n)			All-age mortality			Age-standardized mortality (per 100000)			
Location	Number in 1992	Number in 2021	Percent change of numbers, 1992-2021, %	Rate in 1992, per 100000	Rate in 2021, per 100000	Percent change of rate, 1992-2021, %	Rate in 1992	Rate in 2021	Percent change of numbers, 1992-2021, %	Net drift of mortality, % per year
Denmark	353 (327 to 376)	169 (147 to 187)	-52.1	13.46 (12.46 to 14.36)	5.74 (5.01 to 6.35)	-57.4	8.46 (7.89 to 9.02)	2.88 (2.58 to 3.15)	-66	-3.07 (-4.08 to -2.04)
Estonia	109 (100 to 119)	65 (56 to 75)	-40.4	13.25 (12.14 to 14.49)	9.41 (8.04 to 10.88)	-29	9.06 (8.33 to 9.92)	4.56 (3.88 to 5.28)	-49.7	-2.27 (-3.93 to -0.58)
Finland	114 (103 to 124)	81 (69 to 91)	-28.9	4.38 (3.96 to 4.77)	2.88 (2.45 to 3.25)	-34.2	2.69 (2.43 to 2.93)	1.35 (1.19 to 1.5)	-49.8	-2.18 (-3.65 to -0.70)
France	2020 (1866 to 2167)	1745 (1489 to 1957)	-13.6	6.77 (6.26 to 7.27)	5.1 (4.35 to 5.72)	-24.7	4.4 (4.11 to 4.7)	2.42 (2.16 to 2.67)	-45	-1.77 (-2.12 to -1.42)
Germany	4107 (3787 to 4390)	2301 (2026 to 2545)	-44	9.87 (9.1 to 10.55)	5.37 (4.73 to 5.94)	-45.6	5.74 (5.36 to 6.11)	2.61 (2.37 to 2.83)	-54.5	-2.53 (-2.82 to -2.24)

	Deaths number (n)			All-age mortality			Age-standardized mortality (per 100000)			
Location	Number in 1992	Number in 2021	Percent change of numbers, 1992-2021, %	Rate in 1992, per 100000	Rate in 2021, per 100000	Percent change of rate, 1992-2021, %	Rate in 1992	Rate in 2021	Percent change of numbers, 1992-2021, %	Net drift of mortality, % per year
Greece	361 (336 to 381)	326 (288 to 356)	-9.7	6.76 (6.29 to 7.13)	6.24 (5.5 to 6.8)	-7.7	4.54 (4.24 to 4.78)	2.78 (2.53 to 2.99)	-38.8	-1.47 (-2.43 to -0.51)
Hungary	656 (568 to 752)	450 (380 to 529)	-31.4	12.15 (10.53 to 13.93)	8.96 (7.56 to 10.54)	-26.3	8.35 (7.16 to 9.64)	4.73 (3.94 to 5.63)	-43.4	-2.20 (-3.08 to -1.30)
Iceland	6 (5 to 6)	5 (4 to 6)	-16.7	4.54 (4.07 to 4.95)	3.04 (2.58 to 3.42)	-33	3.9 (3.54 to 4.26)	1.86 (1.61 to 2.09)	-52.3	-2.11 (-7.57 to 3.67)
Ireland	97 (89 to 105)	83 (72 to 93)	-14.4	5.36 (4.88 to 5.81)	3.31 (2.88 to 3.71)	-38.2	4.59 (4.2 to 4.96)	2.15 (1.9 to 2.4)	-53.2	-2.88 (-4.08 to -1.66)
Israel	95 (87 to 103)	127 (110 to 142)	33.7	3.6 (3.3 to 3.9)	2.65 (2.28 to 2.96)	-26.4	3.53 (3.23 to 3.81)	1.97 (1.73 to 2.19)	-44.2	-1.65 (-3.02 to -0.25)

	Deaths number (n)			All-age mortality			Age-standardized mortality (per 100000)			
Location	Number in 1992	Number in 2021	Percent change of numbers, 1992-2021, %	Rate in 1992, per 100000	Rate in 2021, per 100000	Percent change of rate, 1992-2021, %	Rate in 1992	Rate in 2021	Percent change of numbers, 1992-2021, %	Net drift of mortality, % per year
Italy	738 (682 to 774)	1536 (1325 to 1681)	108.1	2.53 (2.34 to 2.65)	5 (4.32 to 5.48)	97.6	1.56 (1.46 to 1.63)	2.13 (1.92 to 2.29)	36.5	2.36 (1.68 to 3.05)
Latvia	158 (145 to 171)	100 (85 to 115)	-36.7	11.2 (10.28 to 12.11)	9.87 (8.38 to 11.38)	-11.9	7.56 (6.95 to 8.17)	4.66 (3.99 to 5.39)	-38.4	-1.60 (-3.08 to -0.09)
Lithuania	228 (212 to 245)	176 (150 to 201)	-22.8	11.7 (10.9 to 12.57)	11.94 (10.22 to 13.63)	2.1	8.6 (8.01 to 9.26)	5.72 (4.87 to 6.55)	-33.5	-1.52 (-2.60 to -0.43)
Luxembourg	12 (11 to 13)	9 (8 to 10)	-25	6.16 (5.72 to 6.58)	2.85 (2.53 to 3.17)	-53.7	4.08 (3.8 to 4.34)	1.64 (1.47 to 1.82)	-59.8	-2.14 (-5.58 to 1.44)
Malta	8 (7 to 8)	8 (7 to 9)	0	4.07 (3.68 to 4.45)	3.58 (3.09 to 4.13)	-12	3.21 (2.91 to 3.5)	1.74 (1.52 to 1.98)	-45.8	-2.48 (-6.80 to 2.05)

	Deaths number (n)			All-age mortality			Age-standardized mortality (per 100000)			
Location	Number in 1992	Number in 2021	Percent change of numbers, 1992-2021, %	Rate in 1992, per 100000	Rate in 2021, per 100000	Percent change of rate, 1992-2021, %	Rate in 1992	Rate in 2021	Percent change of numbers, 1992-2021, %	Net drift of mortality, % per year
Monaco	1 (1 to 2)	1 (1 to 2)	0	8.32 (5.7 to 11.25)	6.61 (4.88 to 8.78)	-20.6	3.87 (2.68 to 5.31)	2.89 (2.1 to 3.83)	-25.3	-0.71 (-7.98 to 7.13)
Montenegro	19 (15 to 25)	22 (17 to 28)	15.8	5.86 (4.65 to 7.74)	7.11 (5.55 to 8.85)	21.3	5.21 (4.15 to 6.93)	4.5 (3.51 to 5.58)	-13.6	-0.58 (-3.33 to 2.25)
Netherlands	371 (339 to 397)	311 (278 to 349)	-16.2	4.84 (4.43 to 5.19)	3.59 (3.21 to 4.02)	-25.8	3.3 (3.04 to 3.54)	1.85 (1.67 to 2.07)	-43.9	-2.00 (-2.90 to -1.10)
North Macedonia	82 (69 to 97)	92 (68 to 120)	12.2	8.28 (6.96 to 9.8)	8.57 (6.34 to 11.19)	3.5	7.97 (6.69 to 9.42)	5.64 (4.2 to 7.33)	-29.2	-0.87 (-2.64 to 0.94)
Norway	174 (163 to 183)	144 (126 to 156)	-17.2	8.04 (7.5 to 8.43)	5.37 (4.7 to 5.83)	-33.2	5.23 (4.96 to 5.47)	2.86 (2.6 to 3.07)	-45.3	-2.08 (-3.18 to -0.96)

	Deaths number (n)			All-age mortality			Age-standardized mortality (per 100000)			
Location	Number in 1992	Number in 2021	Percent change of numbers, 1992-2021, %	Rate in 1992, per 100000	Rate in 2021, per 100000	Percent change of rate, 1992-2021, %	Rate in 1992	Rate in 2021	Percent change of numbers, 1992-2021, %	Net drift of mortality, % per year
Poland	2772 (2672 to 2866)	1958 (1735 to 2162)	-29.4	14.08 (13.57 to 14.56)	9.92 (8.79 to 10.96)	-29.5	11.16 (10.78 to 11.54)	5.17 (4.58 to 5.72)	-53.7	-2.95 (-3.32 to -2.58)
Portugal	462 (424 to 500)	359 (314 to 401)	-22.3	8.8 (8.08 to 9.53)	6.44 (5.63 to 7.2)	-26.8	6.26 (5.77 to 6.75)	2.95 (2.64 to 3.23)	-52.9	-2.62 (-3.52 to -1.72)
Republic of Moldova	259 (243 to 276)	202 (181 to 227)	-22	11.14 (10.46 to 11.88)	10.74 (9.62 to 12.07)	-3.6	9.87 (9.24 to 10.49)	6.49 (5.79 to 7.29)	-34.2	-1.49 (-2.78 to -0.18)
Romania	1972 (1805 to 2121)	1841 (1620 to 2079)	-6.6	16.67 (15.25 to 17.93)	18.92 (16.65 to 21.36)	13.5	13.54 (12.3 to 14.59)	10.45 (9.14 to 11.9)	-22.8	-1.22 (-1.65 to -0.79)

	Deaths number (n)			All-age mortality			Age-standardized mortality (per 100000)			
Location	Number in 1992	Number in 2021	Percent change of numbers, 1992-2021, %	Rate in 1992, per 100000	Rate in 2021, per 100000	Percent change of rate, 1992-2021, %	Rate in 1992	Rate in 2021	Percent change of numbers, 1992-2021, %	Net drift of mortality, % per year
Russian Federation	7202 (6961 to 7450)	7491 (6660 to 8246)	4	8.94 (8.64 to 9.25)	9.68 (8.6 to 10.65)	8.3	6.35 (6.15 to 6.57)	6.14 (5.44 to 6.8)	-3.3	-0.73 (-0.92 to -0.54)
San Marino	0 (0 to 0)	0 (0 to 0)	0	2.79 (2.13 to 3.54)	1.91 (1.19 to 2.86)	-31.5	1.79 (1.38 to 2.28)	0.89 (0.55 to 1.39)	-50.3	-1.98 (-9.15 to 5.76)
Serbia	713 (534 to 931)	596 (466 to 756)	-16.4	14.47 (10.85 to 18.91)	13.33 (10.43 to 16.92)	-7.9	12.03 (9.1 to 15.66)	7.54 (5.88 to 9.56)	-37.3	-1.84 (-2.59 to -1.08)
Slovakia	250 (207 to 299)	231 (172 to 302)	-7.6	9.18 (7.61 to 10.97)	8.33 (6.19 to 10.87)	-9.3	7.58 (6.33 to 8.97)	4.83 (3.61 to 6.24)	-36.3	-1.32 (-2.53 to -0.09)
Slovenia	70 (64 to 76)	53 (43 to 62)	-24.3	6.88 (6.26 to 7.49)	5.06 (4.17 to 5.97)	-26.5	4.92 (4.48 to 5.34)	2.38 (1.95 to 2.8)	-51.6	-2.15 (-4.03 to -0.23)

	Deaths number (n)			All-age mortality			Age-standardized mortality (per 100000)			
Location	Number in 1992	Number in 2021	Percent change of numbers, 1992-2021, %	Rate in 1992, per 100000	Rate in 2021, per 100000	Percent change of rate, 1992-2021, %	Rate in 1992	Rate in 2021	Percent change of numbers, 1992-2021, %	Net drift of mortality, % per year
Spain	1036 (948 to 1117)	923 (802 to 1028)	-10.9	5.21 (4.77 to 5.62)	3.96 (3.44 to 4.42)	-24	3.57 (3.3 to 3.85)	1.92 (1.72 to 2.12)	-46.2	-1.98 (-2.44 to -1.52)
Sweden	287 (263 to 310)	265 (221 to 301)	-7.7	6.51 (5.97 to 7.04)	5.14 (4.28 to 5.85)	-21	3.68 (3.4 to 3.97)	2.38 (2.03 to 2.72)	-35.3	-1.36 (-2.33 to -0.37)
Switzerland	240 (215 to 263)	141 (117 to 160)	-41.3	6.79 (6.09 to 7.44)	3.16 (2.61 to 3.57)	-53.5	4.16 (3.81 to 4.51)	1.52 (1.33 to 1.7)	-63.5	-3.17 (-4.21 to -2.11)
Ukraine	4330 (4000 to 4677)	1506 (967 to 2147)	-65.2	15.39 (14.21 to 16.62)	6.51 (4.18 to 9.29)	-57.7	10.12 (9.41 to 10.88)	3.56 (2.24 to 5.19)	-64.8	-3.95 (-4.29 to -3.61)

	Deaths number (n)			All-age mortality			Age-standardized mortality (per 100000)			
Location	Number in 1992	Number in 2021	Percent change of numbers, 1992-2021, %	Rate in 1992, per 100000	Rate in 2021, per 100000	Percent change of rate, 1992-2021, %	Rate in 1992	Rate in 2021	Percent change of numbers, 1992-2021, %	Net drift of mortality, % per year
United Kingdom	2453 (2338 to 2518)	1315 (1207 to 1377)	-46.4	8.28 (7.89 to 8.5)	3.81 (3.49 to 3.99)	-54	5.41 (5.23 to 5.52)	2.2 (2.07 to 2.28)	-59.3	-2.50 (-2.81 to -2.19)
