# Supplementary material for: Mortality of Three Major Gynecological Cancers in the European Region: An Age–Period–Cohort Analysis from 1992 to 2021 and Predictions in a 25‑Year Period
Source: Ann Glob Health. 2025 Jun 10;91(1):30. doi: 10.5334/aogh.4688 (PMC12171803; doi:10.5334/aogh.4688)
Supplement: Supplementary Figure 1. — Temporal trends of the numbers of death, all‑age mortality, and age‑standardized mortality for three gynecological cancers (ovarian, uterine, and cervical cancers) among the six WHO regions from 1992 to 2021. [file agh-91-1-4688-s8.pdf]

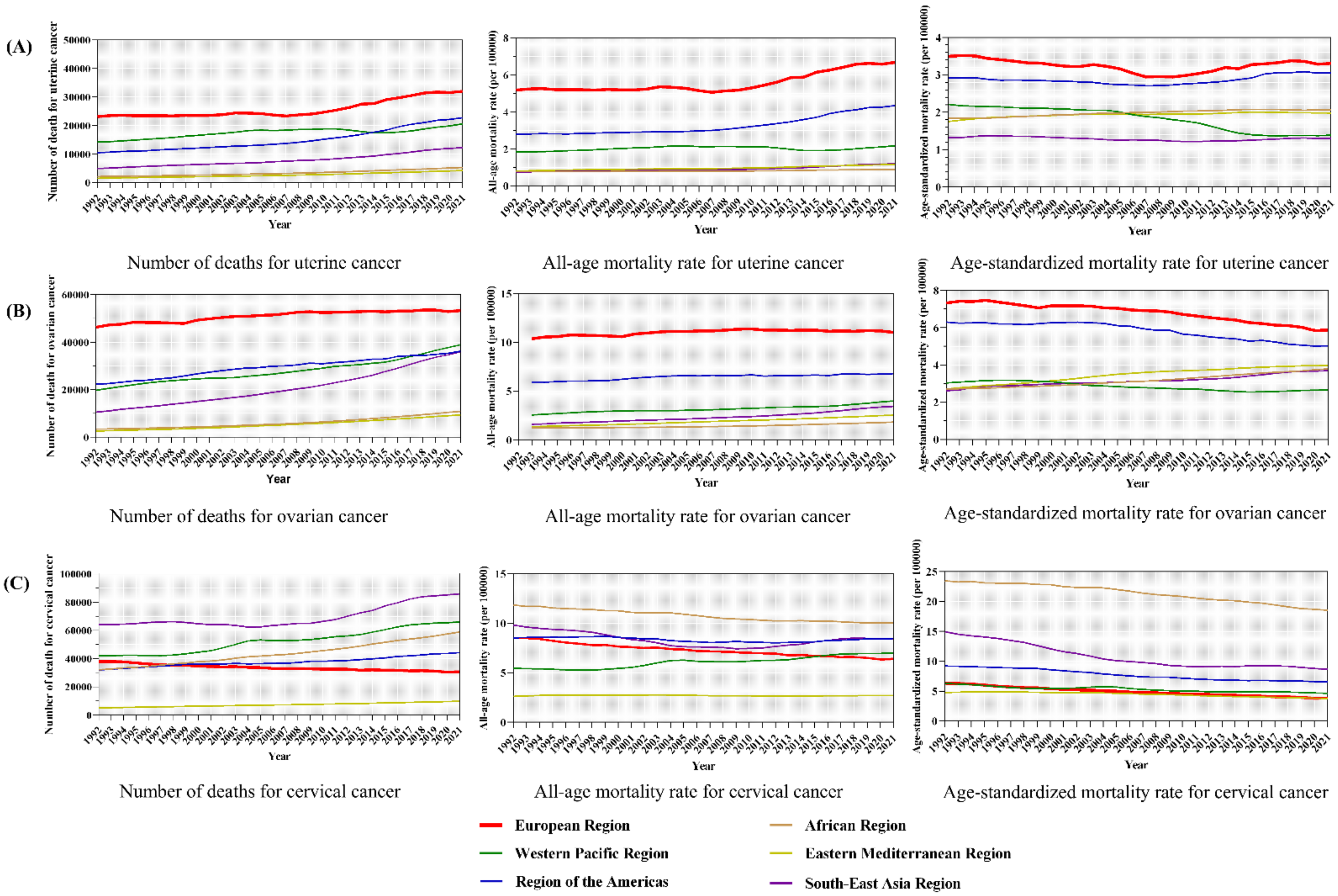

Figure S1. Temporal trends of the numbers of death, all-age mortality, and age-standardized mortality for three gynecological cancers (uterine, cervical, and ovarian cancers) among the six WHO regions from 1992 to 2021.
