# Supplementary material for: Mortality of Three Major Gynecological Cancers in the European Region: An Age–Period–Cohort Analysis from 1992 to 2021 and Predictions in a 25‑Year Period
Source: Ann Glob Health. 2025 Jun 10;91(1):30. doi: 10.5334/aogh.4688 (PMC12171803; doi:10.5334/aogh.4688)
Supplement: Supplementary Figure 6. — The temporal change of the death rate for uterine cancer across age groups in 44 countries within the European Region from 1992 to 2021. [file agh-91-1-4688-s13.pdf]

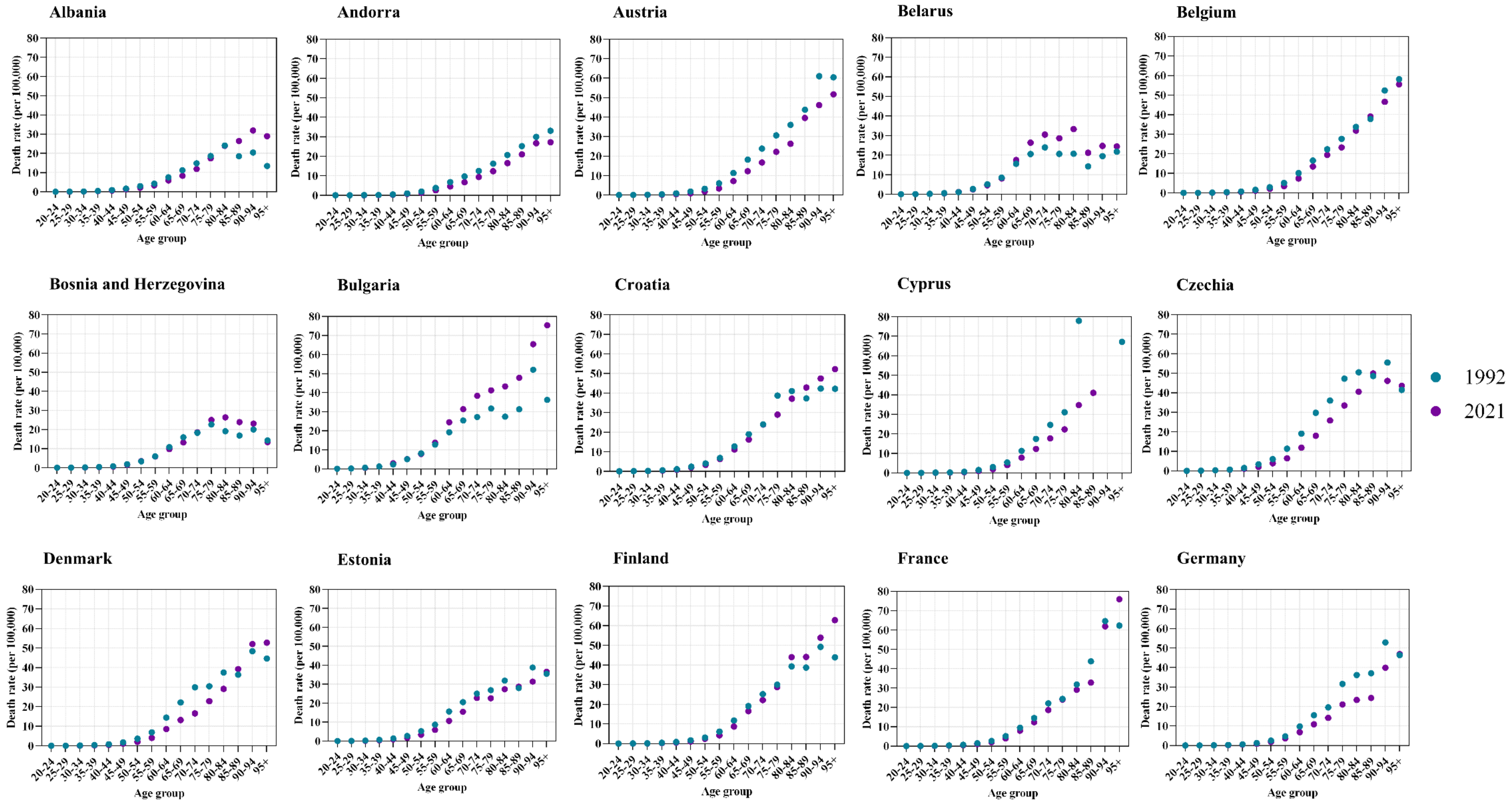

Figure S6. The temporal change of the death rate for uterine cancer across age groups in 44 countries within the European Region from 1992 to 2021.

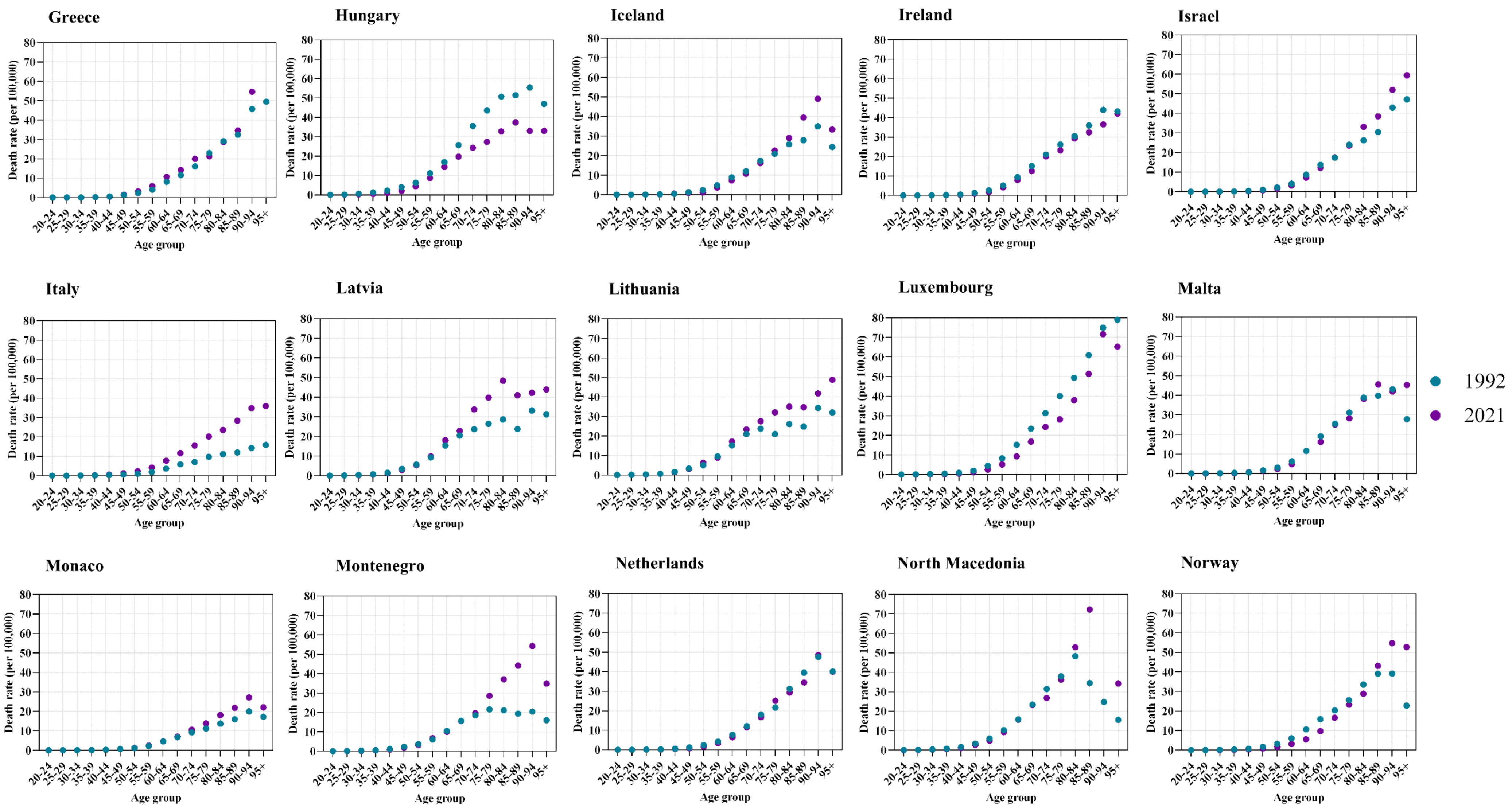

Figure S6 (Continue). The temporal change of the death rate for uterine cancer across age groups in 44 countries within the European Region from 1992 to 2021.

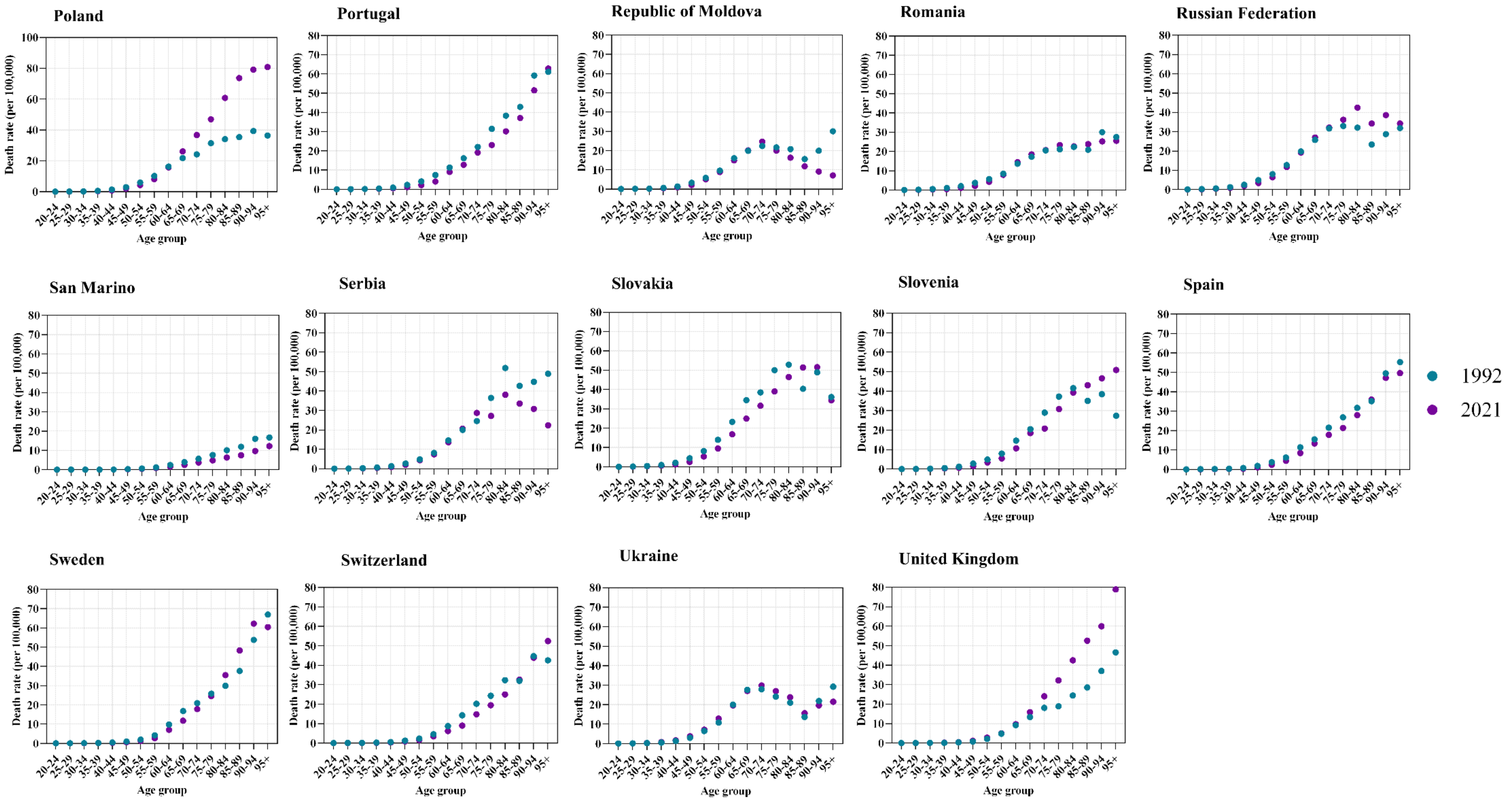

Figure S6 (Continue). The temporal change of the death rate for uterine cancer across age groups in 44 countries within the European Region from 1992 to 2021.
