# Supplementary material for: Mortality of Three Major Gynecological Cancers in the European Region: An Age–Period–Cohort Analysis from 1992 to 2021 and Predictions in a 25‑Year Period
Source: Ann Glob Health. 2025 Jun 10;91(1):30. doi: 10.5334/aogh.4688 (PMC12171803; doi:10.5334/aogh.4688)

Figure S8. The local drift of ovarian cancer deaths in 44 countries within the European Region from 1992 to 2021.

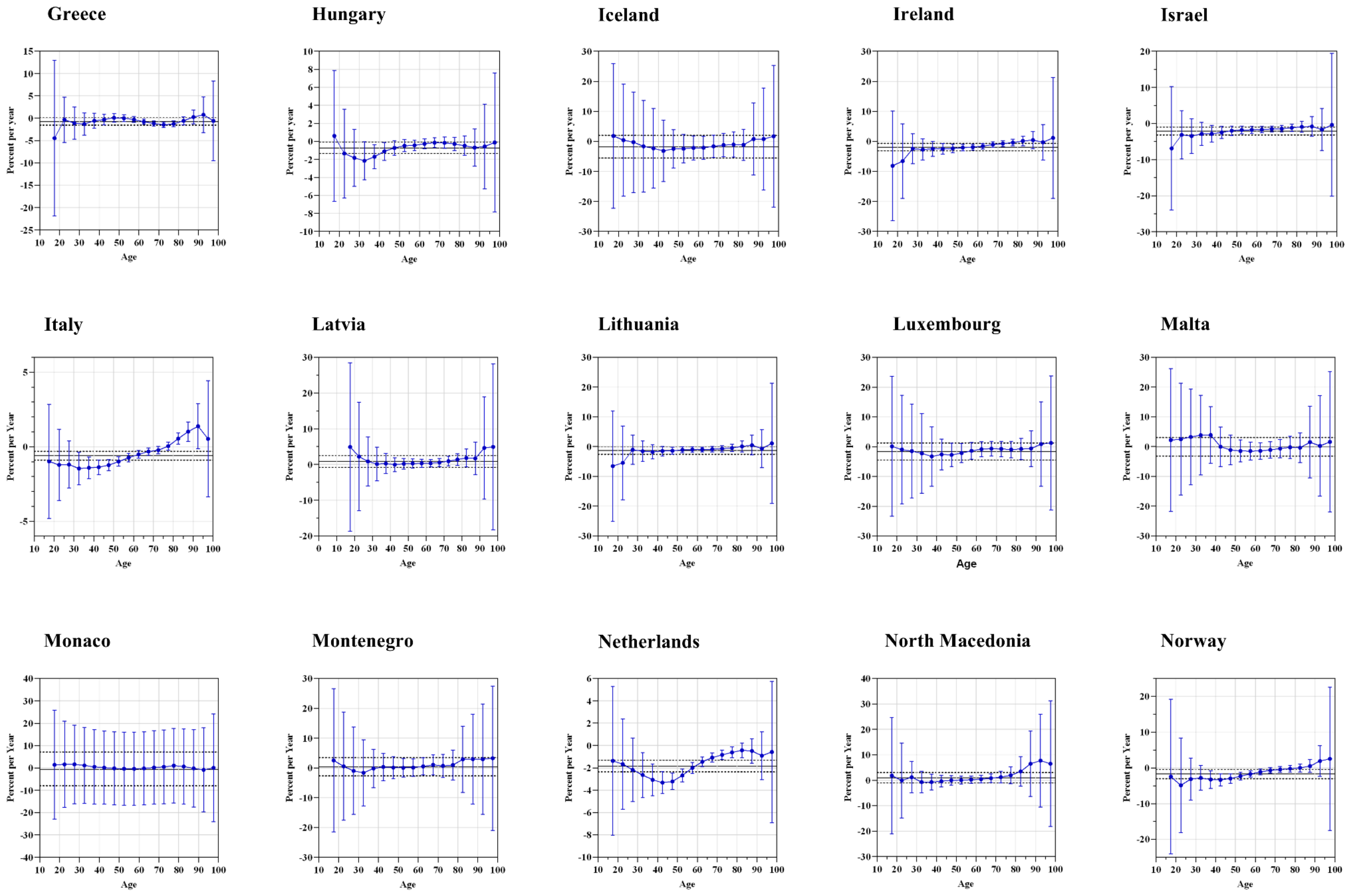

Figure S8 (Continue). The local drift of ovarian cancer deaths in 44 countries within the European Region from 1992 to 2021.

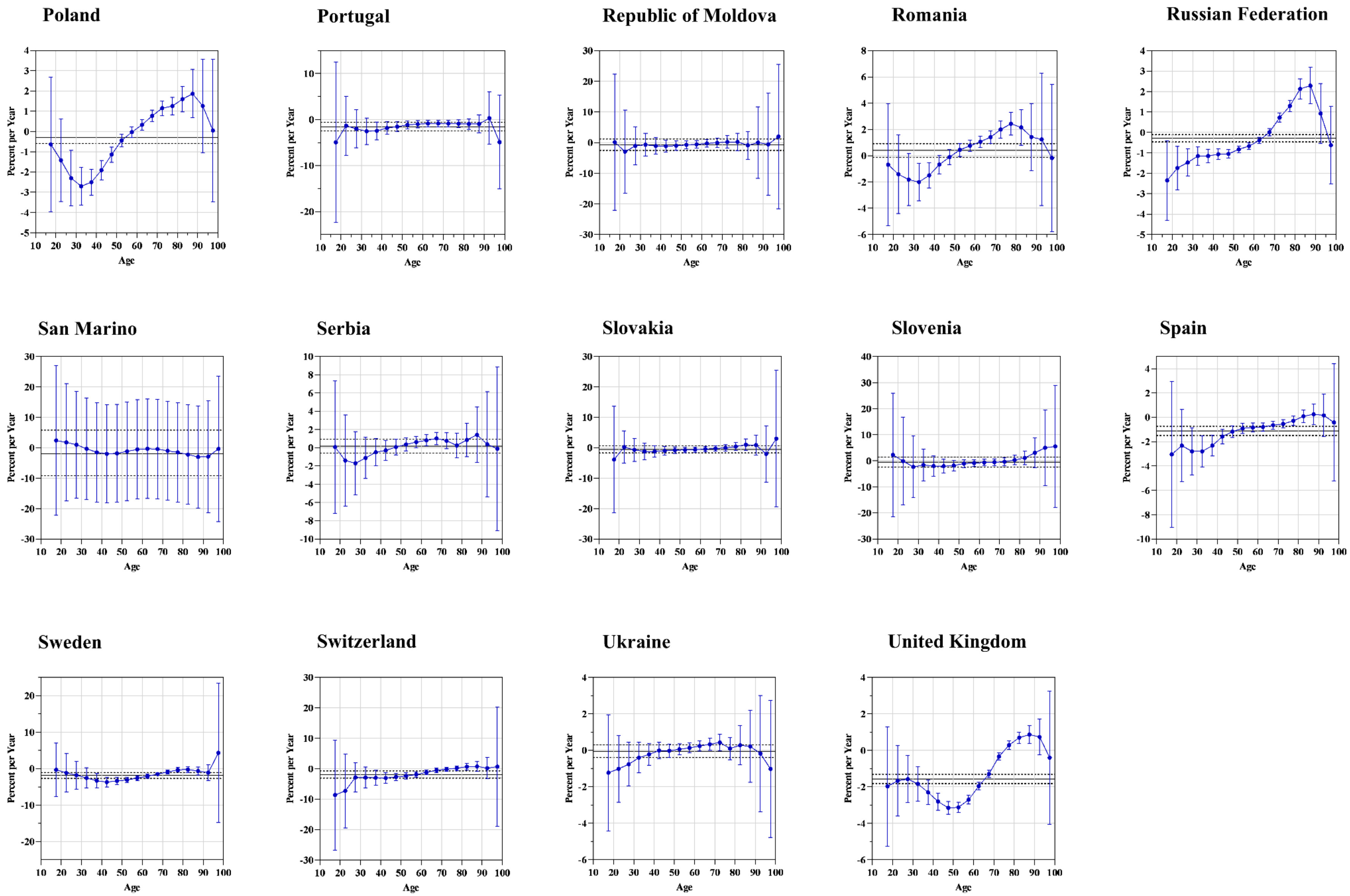

Figure S8 (Continue). The local drift of ovarian cancer deaths in 44 countries within the European Region from 1992 to 2021.

Albania

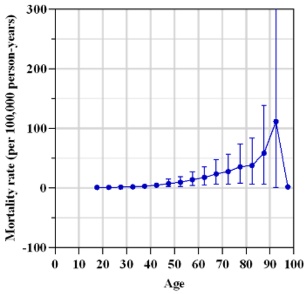

Andorra

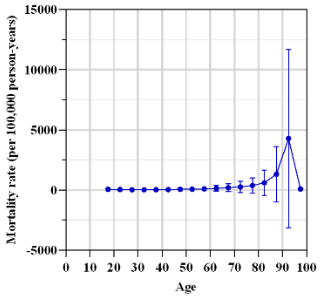

Austria

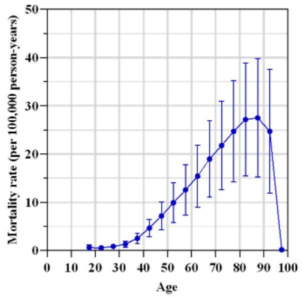

Belarus

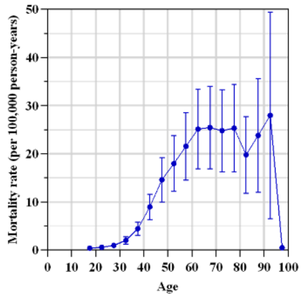

Belgium

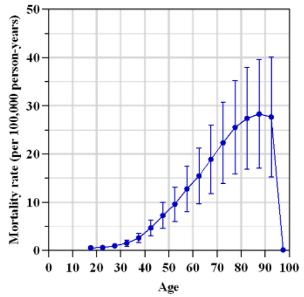

Bosnia and Herzegovina

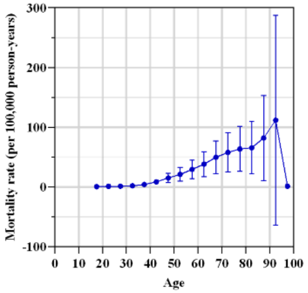

Bulgaria

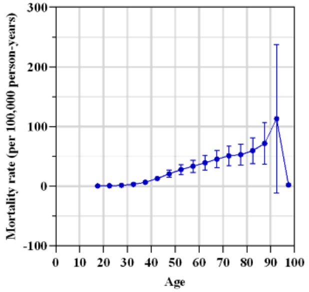

Croatia

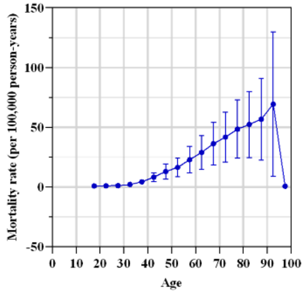

Cyprus

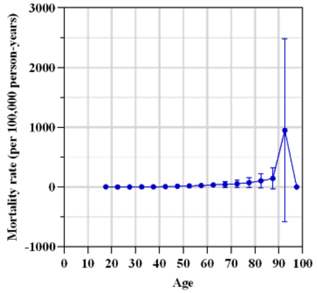

Czechia

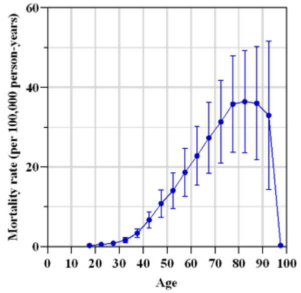

Denmark

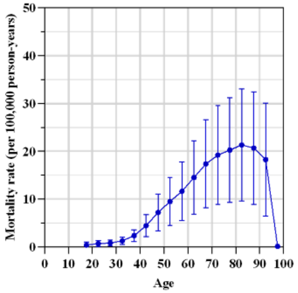

Estonia

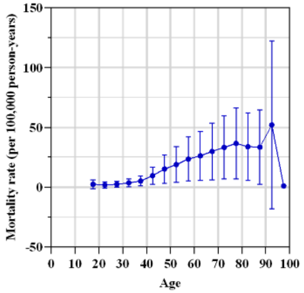

Finland

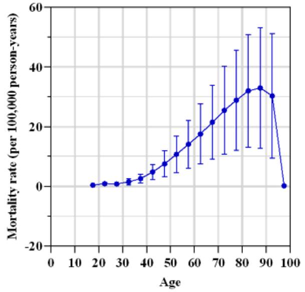

France

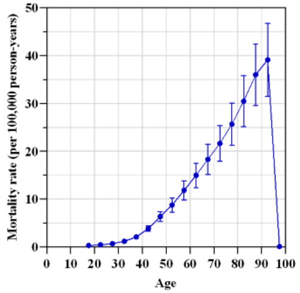

Germany

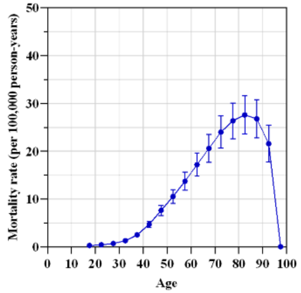

Supplement: Supplementary Figure 8. — The local drift of ovarian cancer deaths in 44 countries within the European Region from 1992 to 2021. [file agh-91-1-4688-s15.pdf]
