# Supplementary material for: Mortality of Three Major Gynecological Cancers in the European Region: An Age–Period–Cohort Analysis from 1992 to 2021 and Predictions in a 25‑Year Period
Source: Ann Glob Health. 2025 Jun 10;91(1):30. doi: 10.5334/aogh.4688 (PMC12171803; doi:10.5334/aogh.4688)

Figure S17. The age effects of cervical cancer deaths in 44 countries within the European Region from 1992 to 2021.

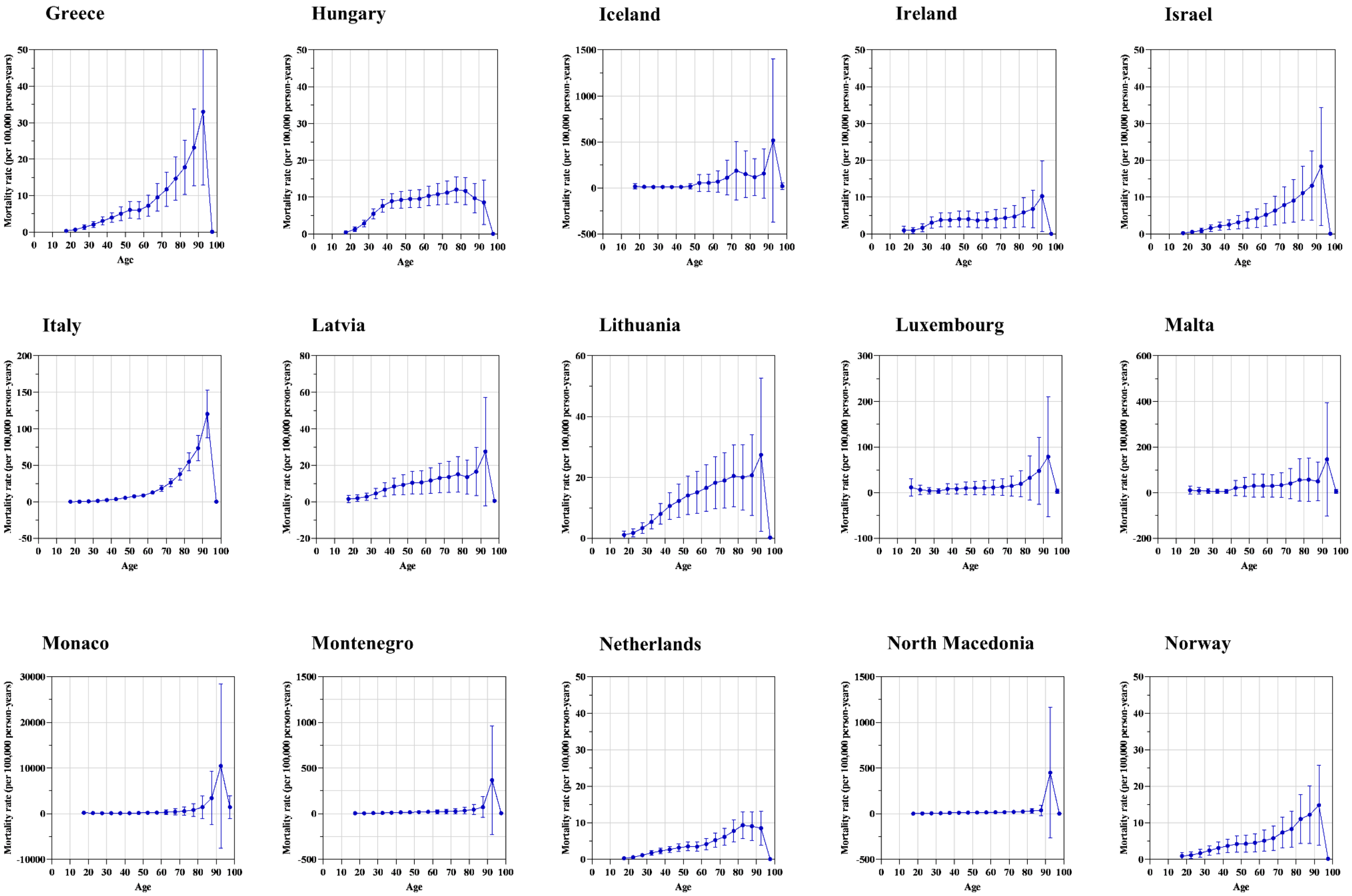

Figure S17 (Continue). The age effects of cervical cancer deaths in 44 countries within the European Region from 1992 to 2021.

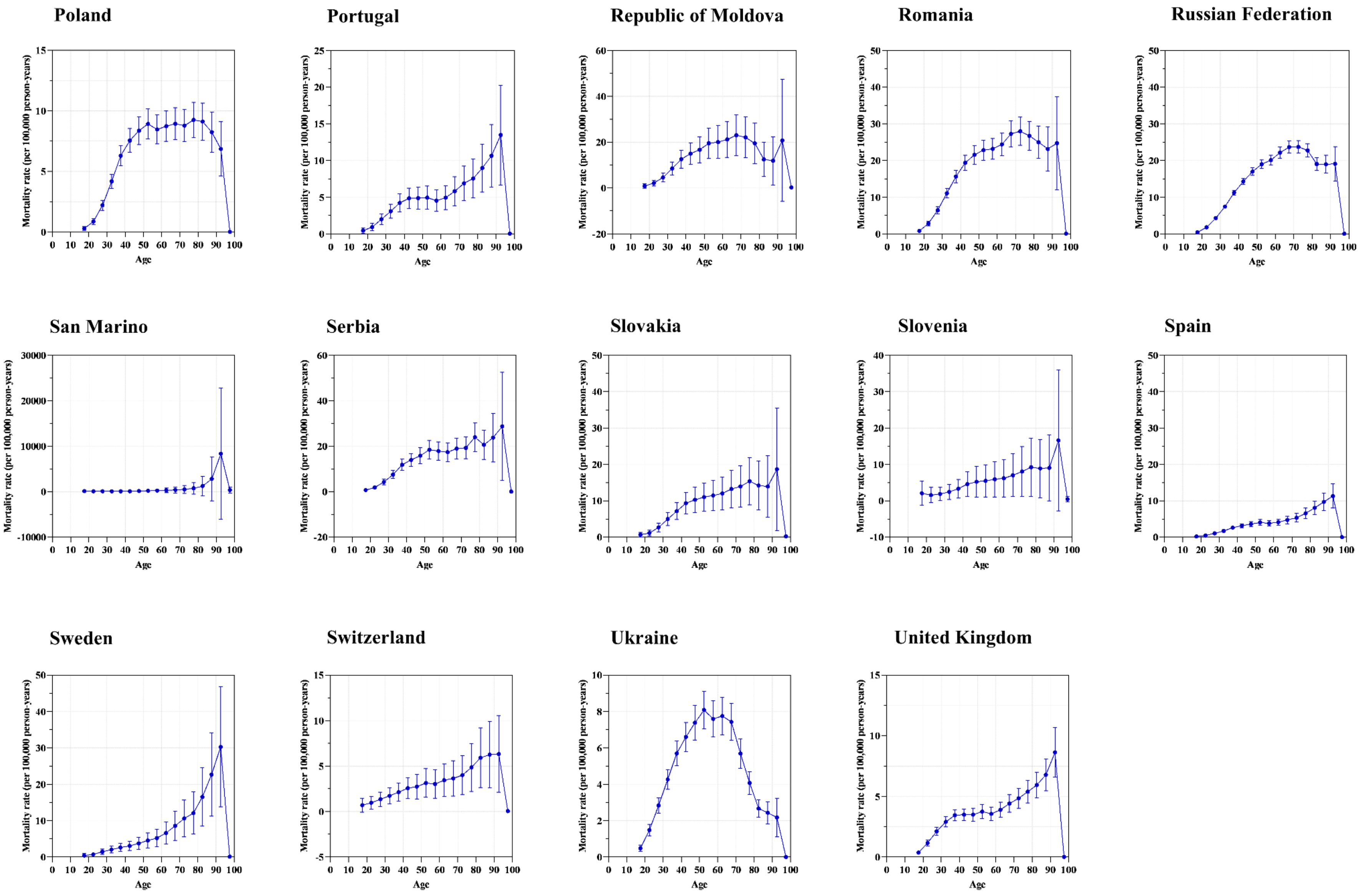

Figure S17 (Continue). The age effects of cervical cancer deaths in 44 countries within the European Region from 1992 to 2021.

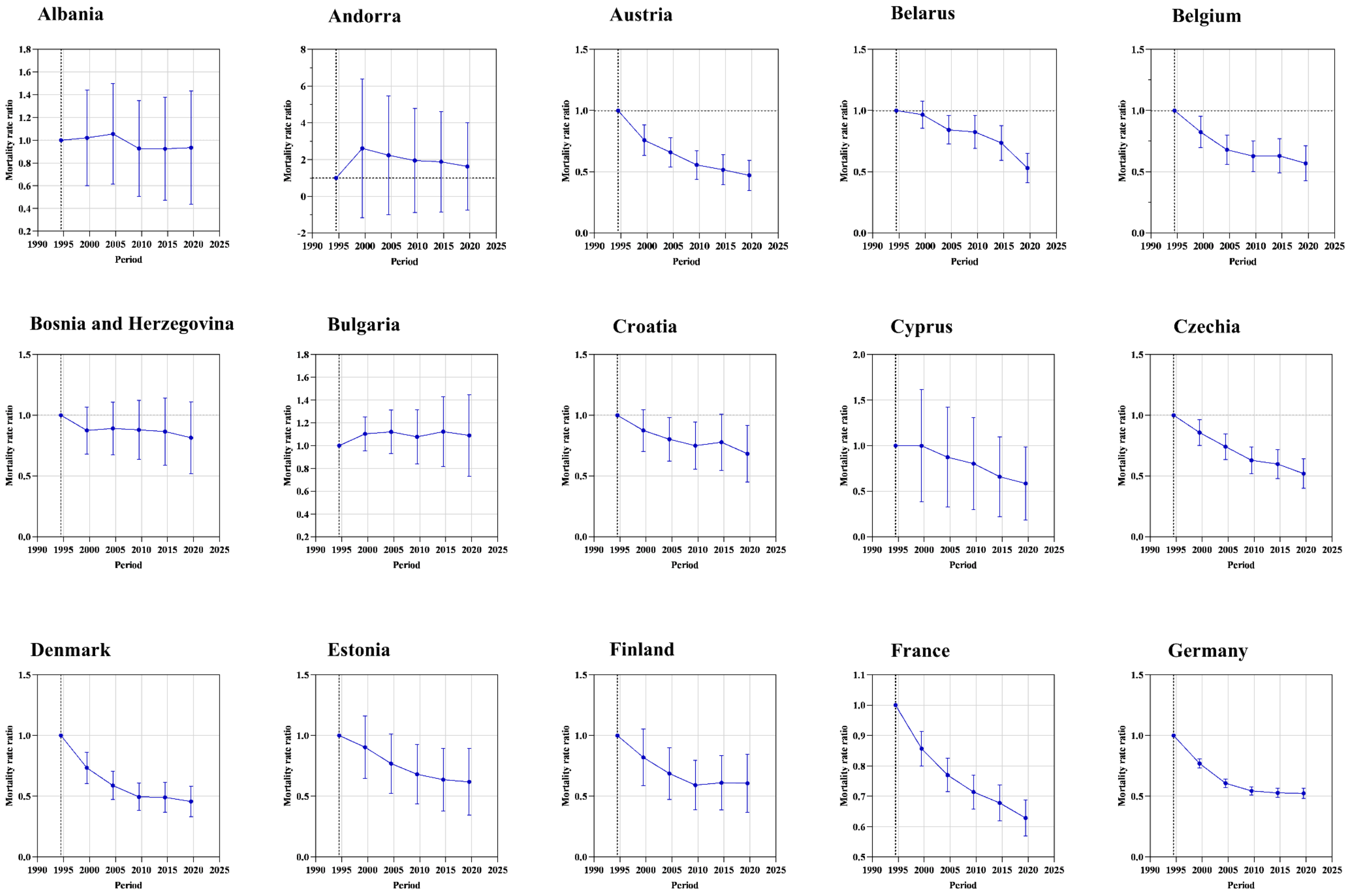

Supplement: Supplementary Figure 17. — The age effects of cervical cancer deaths in 44 countries within the European Region from 1992 to 2021. [file agh-91-1-4688-s24.pdf]
