# Supplementary material for: Mortality of Three Major Gynecological Cancers in the European Region: An Age–Period–Cohort Analysis from 1992 to 2021 and Predictions in a 25‑Year Period
Source: Ann Glob Health. 2025 Jun 10;91(1):30. doi: 10.5334/aogh.4688 (PMC12171803; doi:10.5334/aogh.4688)

Figure S19. The cohort effects of cervical cancer deaths in 44 countries within the European Region from 1992 to 2021.

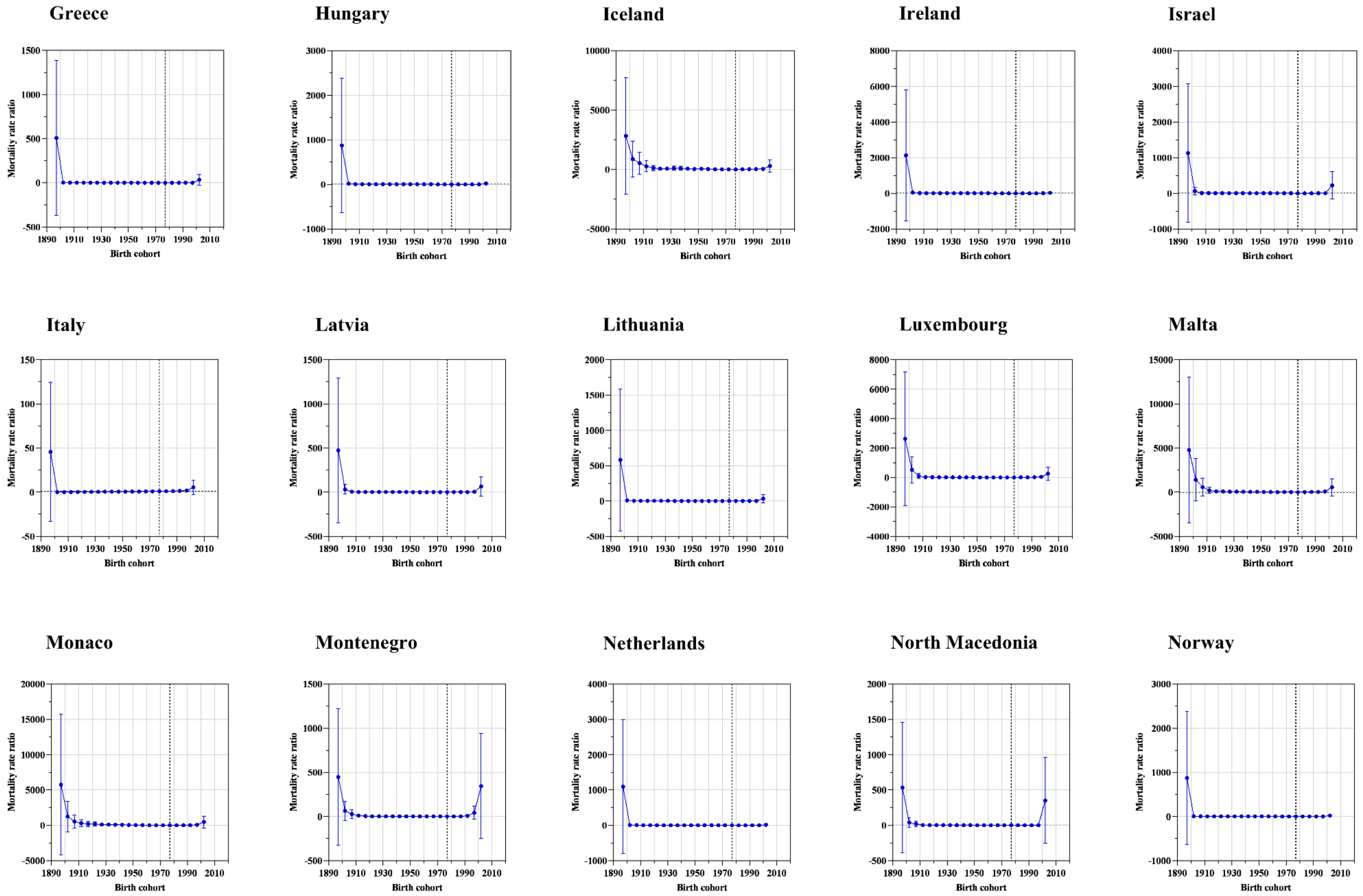

Figure S19 (Continue). The cohort effects of cervical cancer deaths in 44 countries within the European Region from 1992 to 2021.

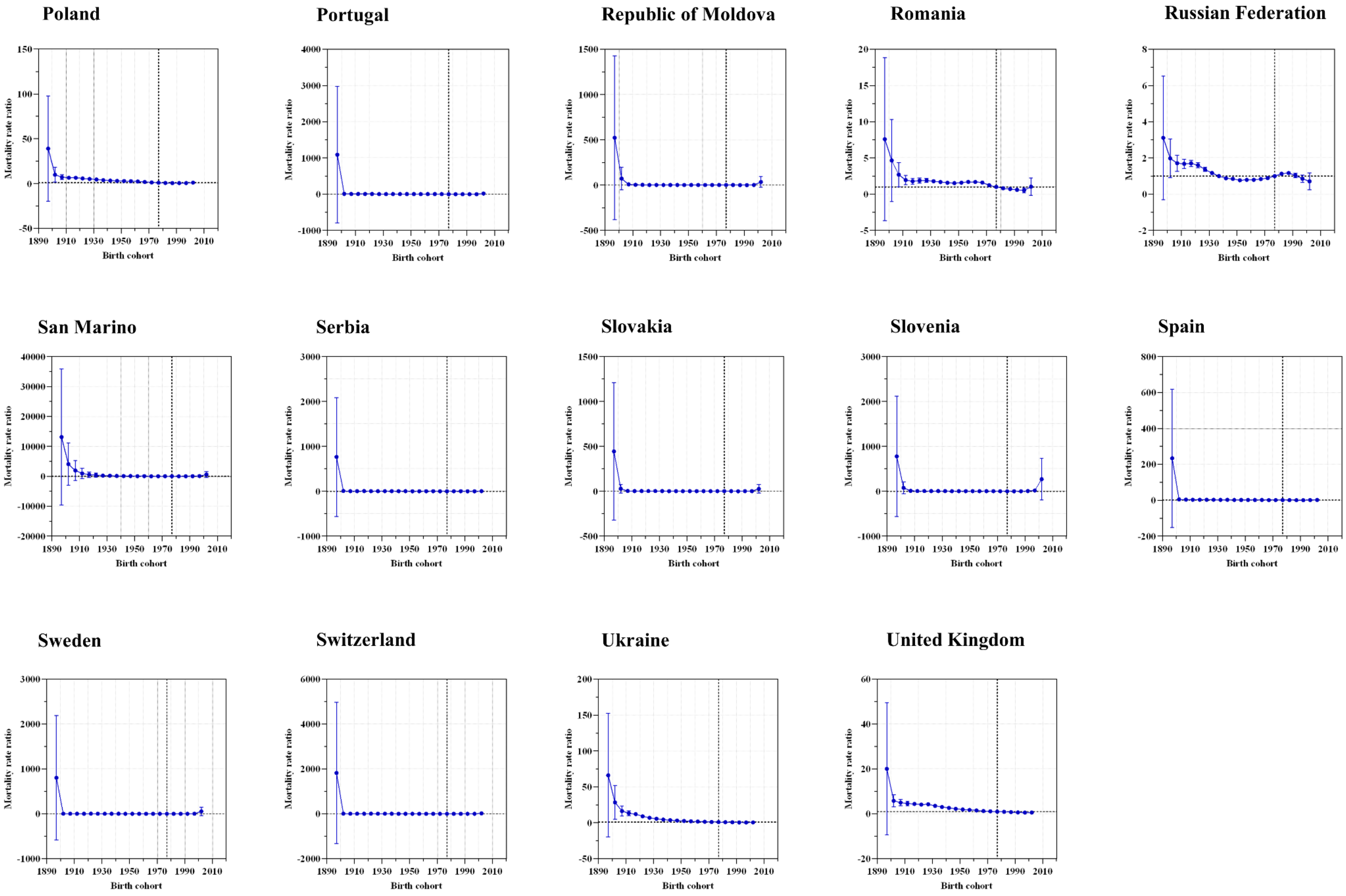
Poland
Portugal
Republic of Moldova
Romania
Russian Federation
San Marino
Serbia
Slovakia
Slovenia
Spain
Sweden
Switzerland
Ukraine
United Kingdom
Mortality rate ratio
Birth cohort

Figure S19 (Continue). The cohort effects of cervical cancer deaths in 44 countries within the European Region from 1992 to 2021.

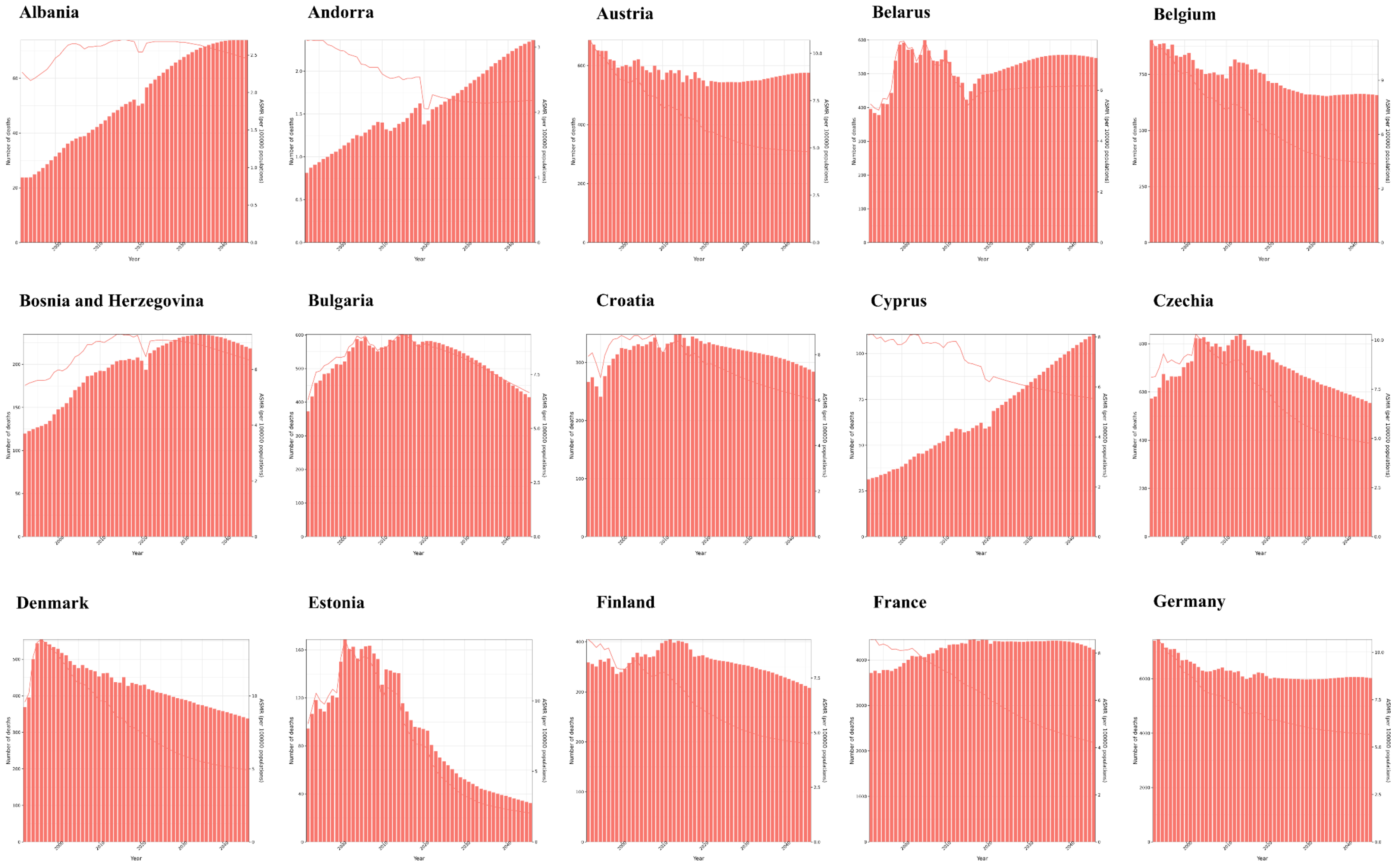

Supplement: Supplementary Figure 19. — The cohort effects of cervical cancer deaths in 44 countries within the European Region from 1992 to 2021. [file agh-91-1-4688-s26.pdf]
