# Supplementary material for: Mortality of Three Major Gynecological Cancers in the European Region: An Age–Period–Cohort Analysis from 1992 to 2021 and Predictions in a 25‑Year Period
Source: Ann Glob Health. 2025 Jun 10;91(1):30. doi: 10.5334/aogh.4688 (PMC12171803; doi:10.5334/aogh.4688)

Figure S20. Observed and predicted ovarian cancer-related death numbers and age-standardized mortality rate in 44 countries within the European Region from 1992 to 2046.

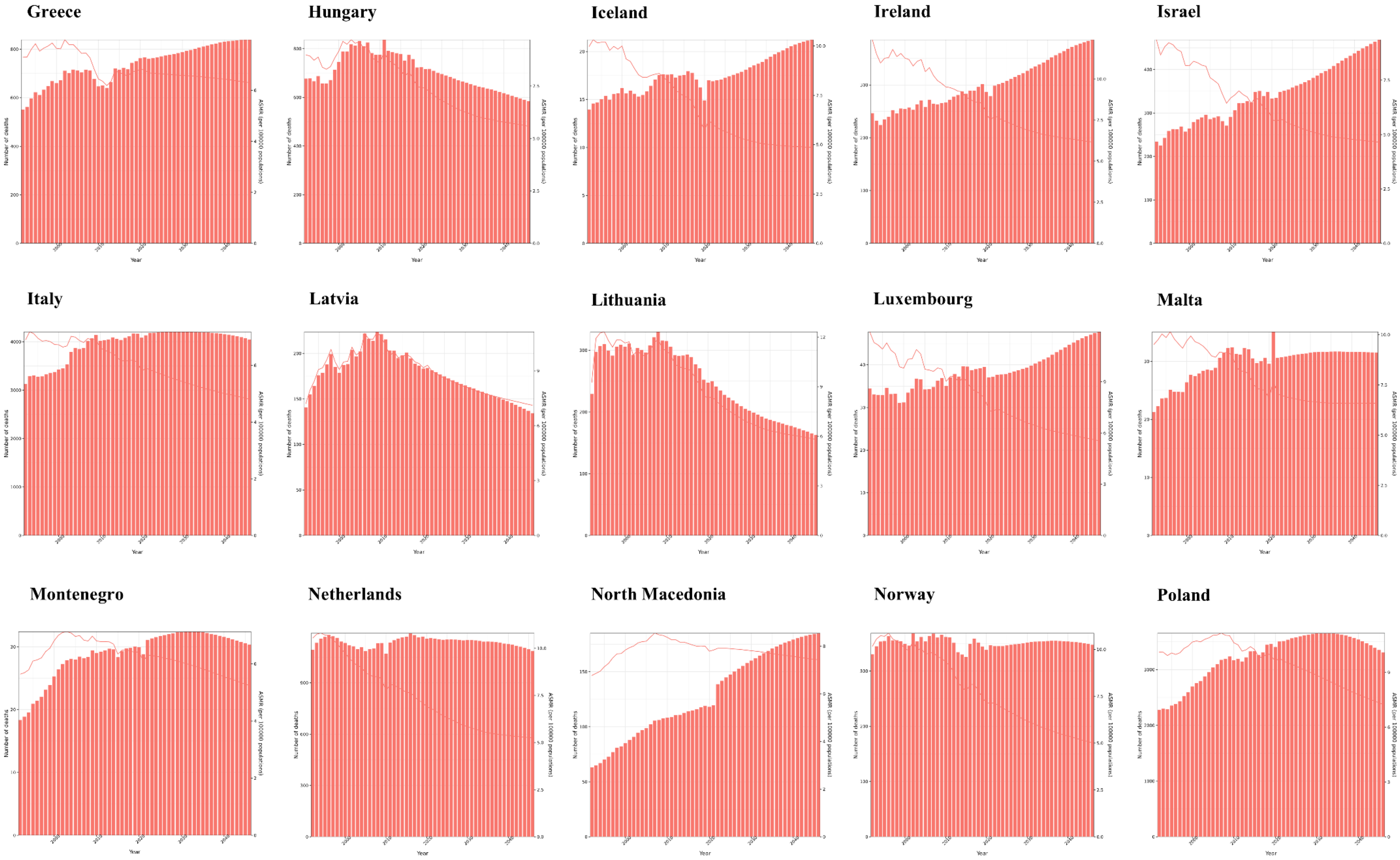

Figure S20 (Continue). Observed and predicted ovarian cancer-related death numbers and age-standardized mortality rate in 44 countries within the European Region from 1992 to 2046.

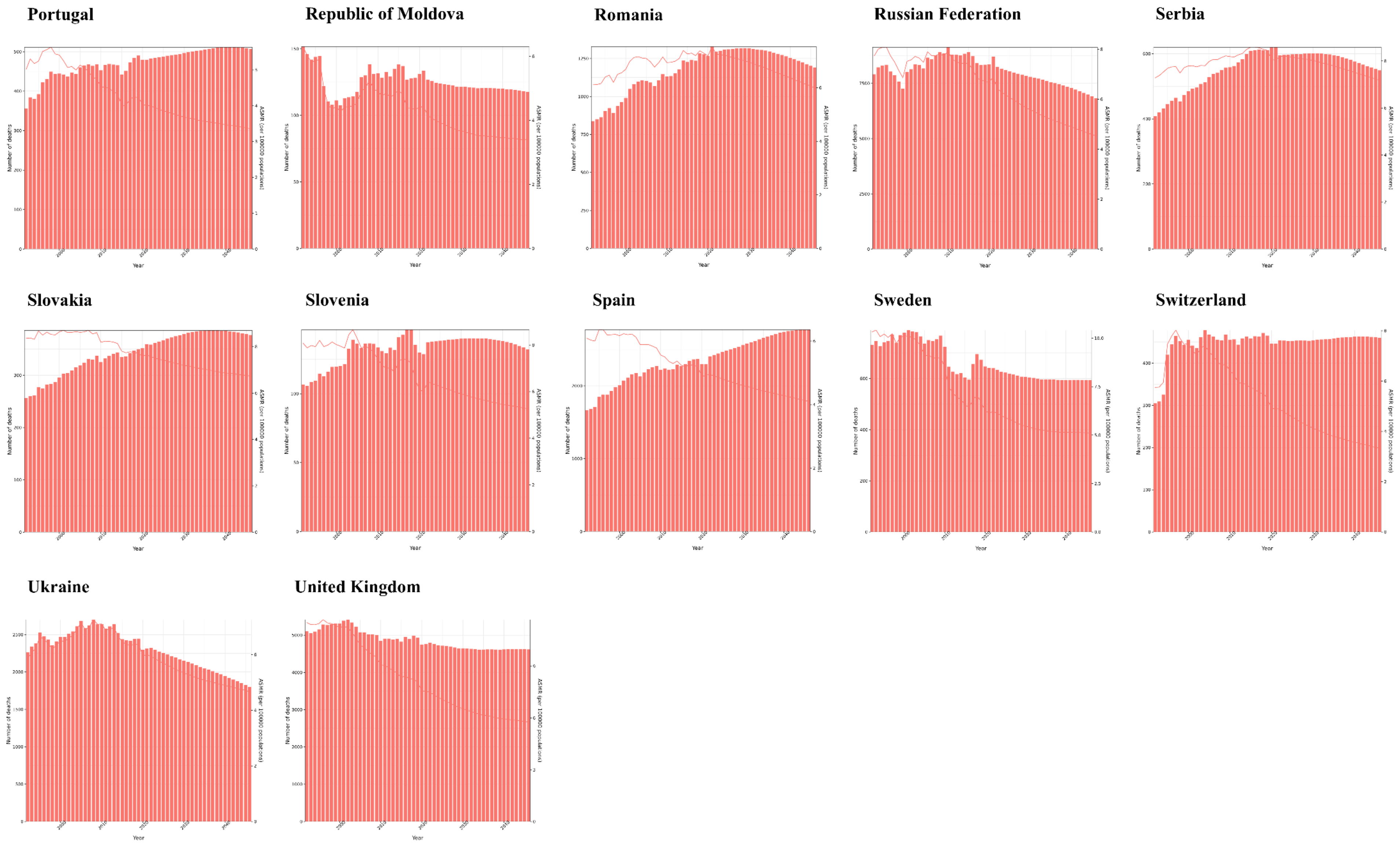

Figure S20 (Continue). Observed and predicted ovarian cancer-related death numbers and age-standardized mortality rate in 44 countries within the European Region from 1992 to 2046.

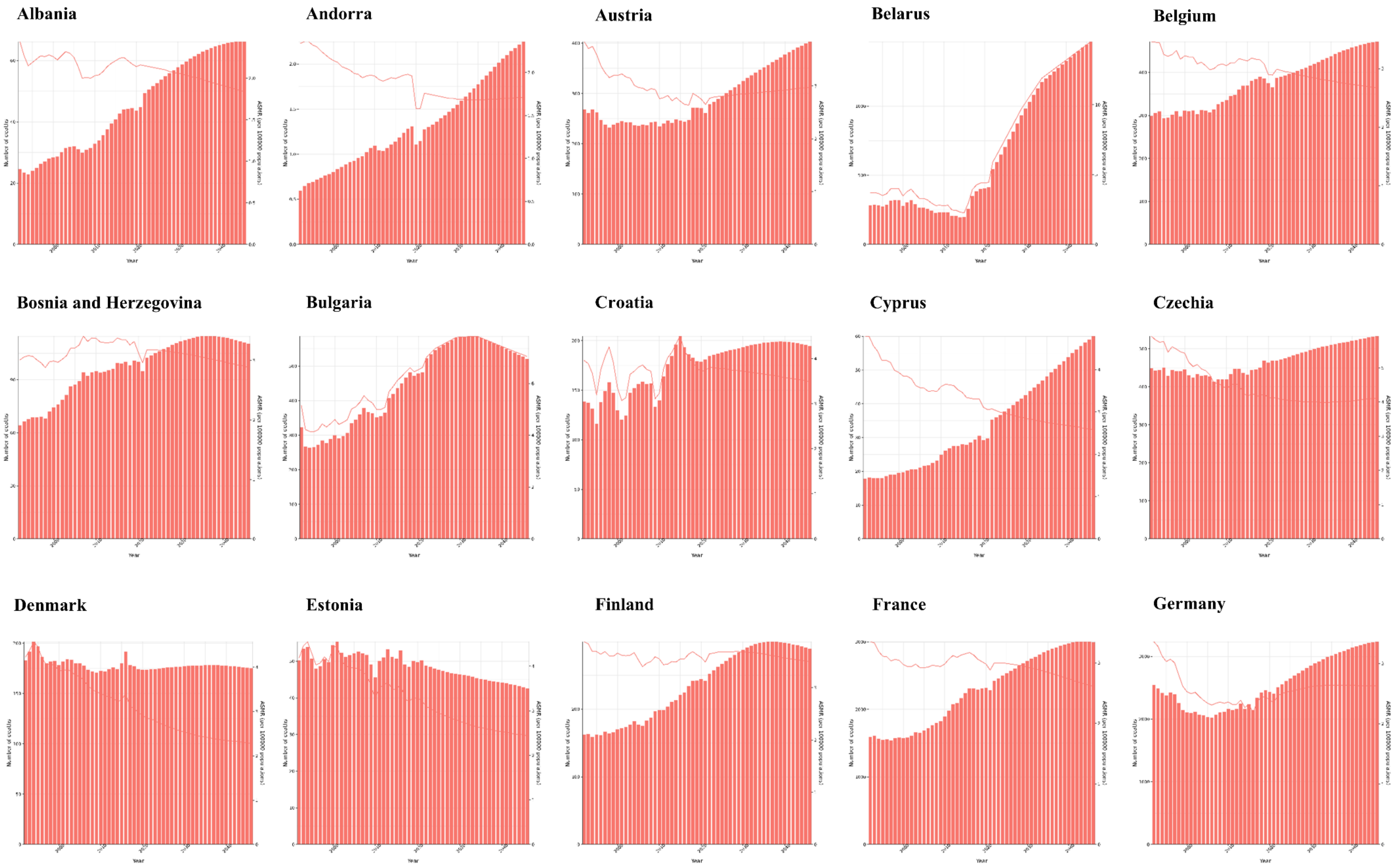

Supplement: Supplementary Figure 20. — Observed and predicted ovarian cancer‑related death numbers and age‑standardized mortality rate in 44 countries within the European Region from 1992 to 2046. [file agh-91-1-4688-s27.pdf]
