# Supplementary material for: Mortality of Three Major Gynecological Cancers in the European Region: An Age–Period–Cohort Analysis from 1992 to 2021 and Predictions in a 25‑Year Period
Source: Ann Glob Health. 2025 Jun 10;91(1):30. doi: 10.5334/aogh.4688 (PMC12171803; doi:10.5334/aogh.4688)

Figure S21. Observed and predicted uterine cancer-related death numbers and age-standardized mortality rate in 44 countries within the European Region from 1992 to 2046.

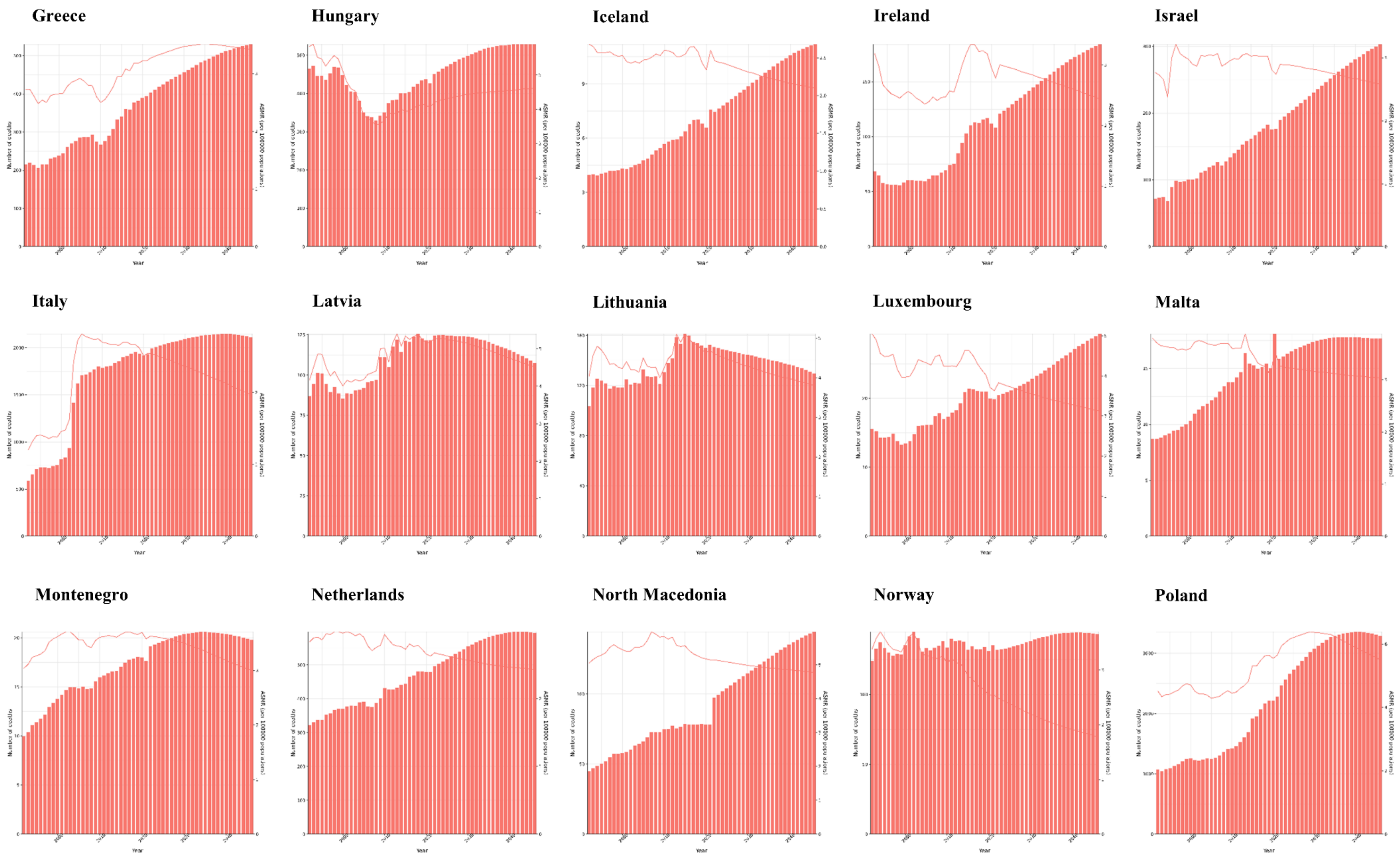

Figure S21 (Continue). Observed and predicted uterine cancer-related death numbers and age-standardized mortality rate in 44 countries within the European Region from 1992 to 2046.

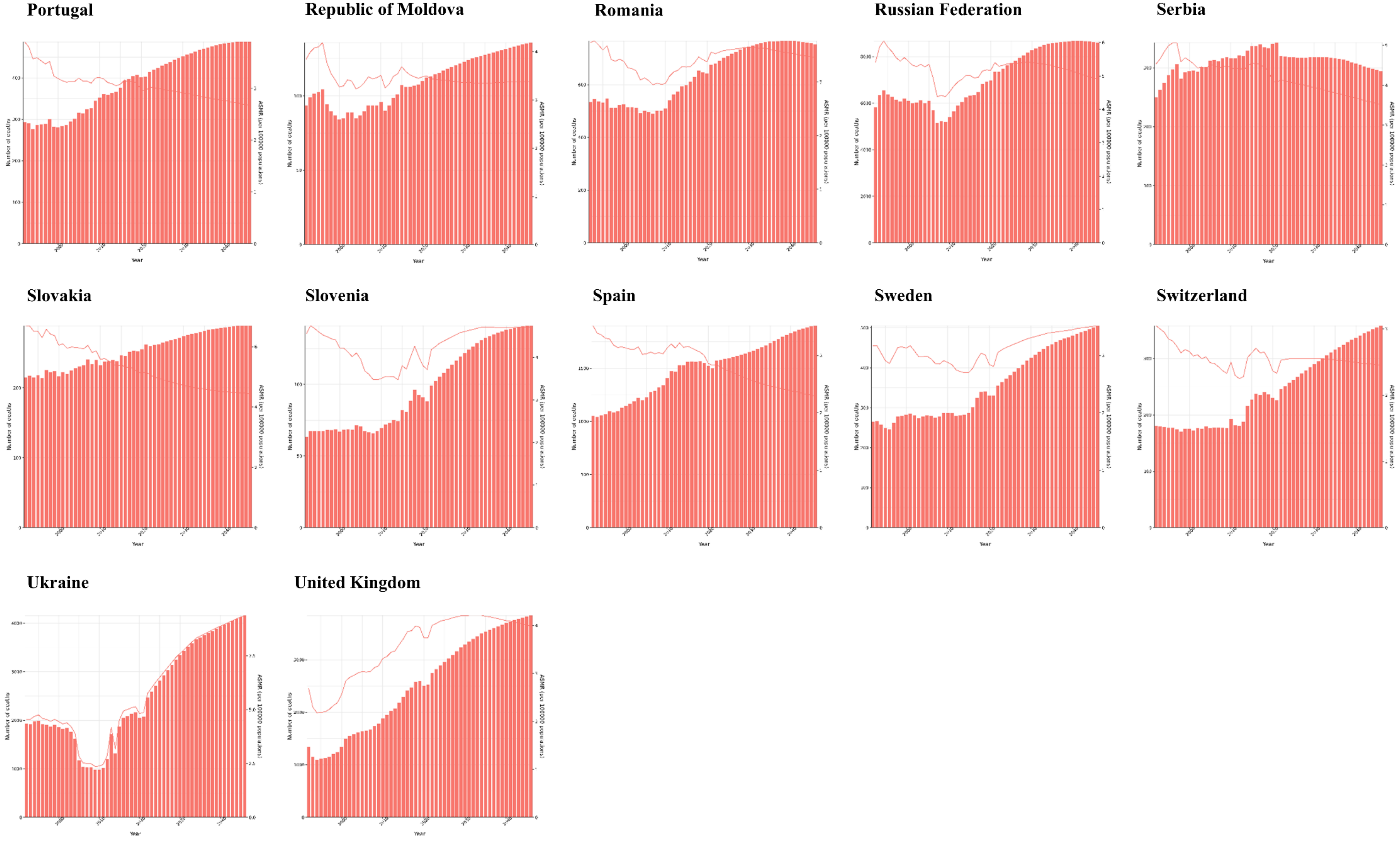

Figure S21 (Continue). Observed and predicted uterine cancer-related death numbers and age-standardized mortality rate in 44 countries within the European Region from 1992 to 2046.

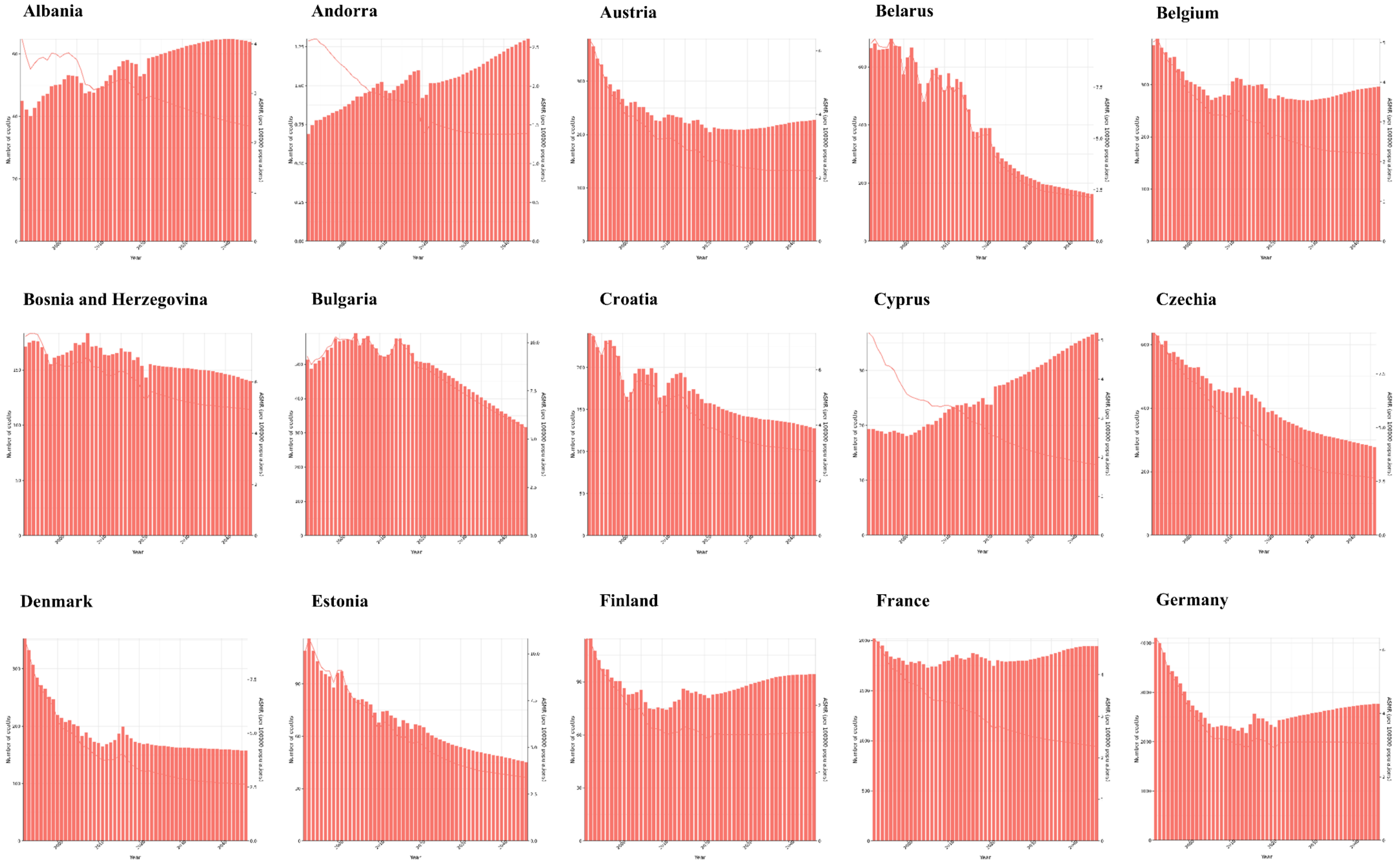

Supplement: Supplementary Figure 21. — Observed and predicted uterine cancer‑related death numbers and age‑standardized mortality rate in 44 countries within the European Region from 1992 to 2046. [file agh-91-1-4688-s28.pdf]
