# Supplementary material for: Mortality of Three Major Gynecological Cancers in the European Region: An Age–Period–Cohort Analysis from 1992 to 2021 and Predictions in a 25‑Year Period
Source: Ann Glob Health. 2025 Jun 10;91(1):30. doi: 10.5334/aogh.4688 (PMC12171803; doi:10.5334/aogh.4688)
Supplement: Supplementary Figure 22. — Observed and predicted cervical cancer‑related death numbers and age‑standardized mortality rate in 44 countries within the European Region from 1992 to 2046. [file agh-91-1-4688-s29.pdf]

Figure S22. Observed and predicted cervical cancer-related death numbers and age-standardized mortality rate in 44 countries within the European Region from 1992 to 2046.

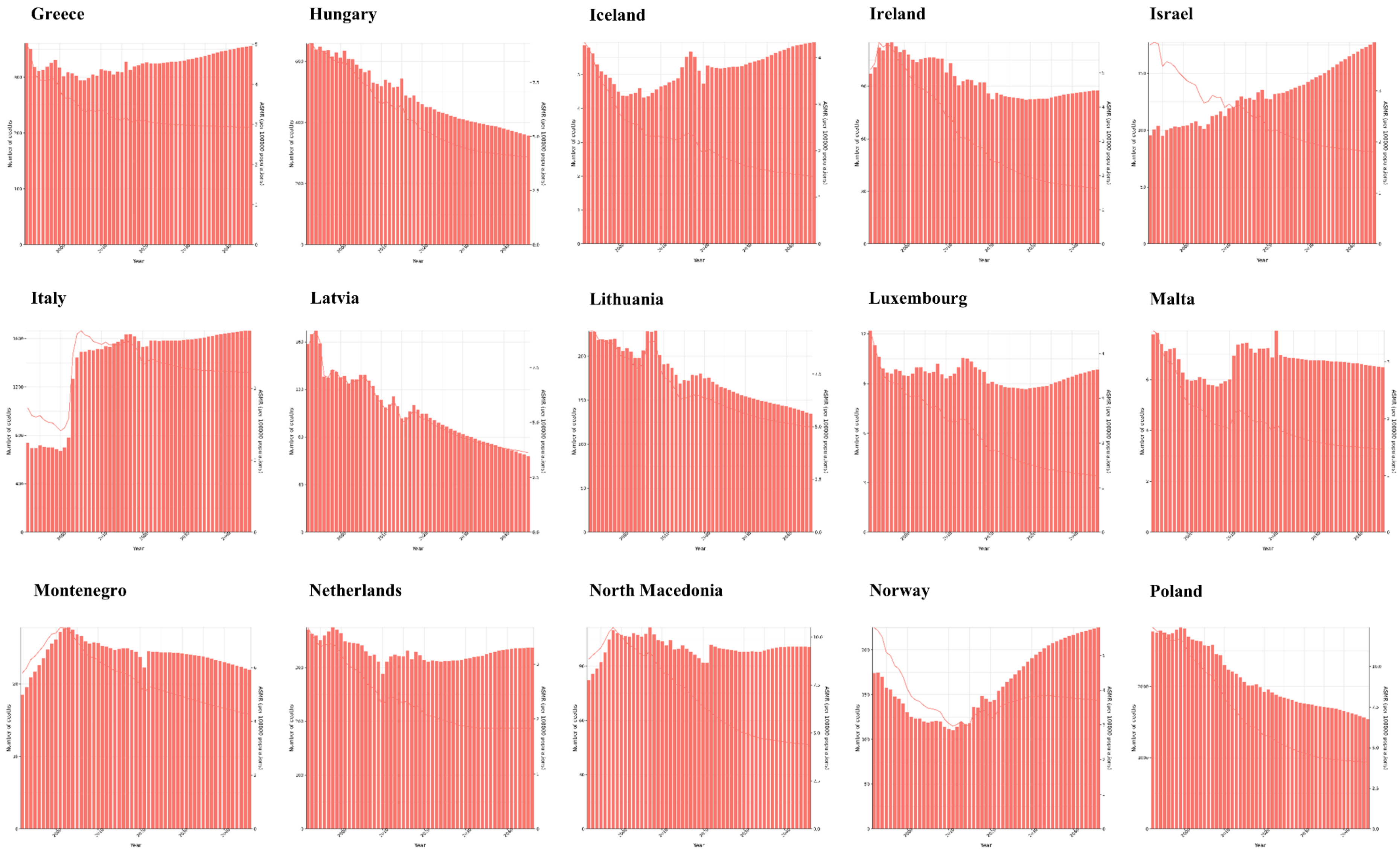

Figure S22 (Continue). Observed and predicted cervical cancer-related death numbers and age-standardized mortality rate in 44 countries within the European Region from 1992 to 2046.

Portugal

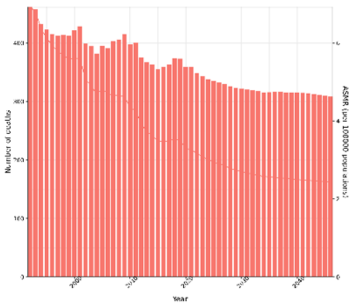

Republic of Moldova

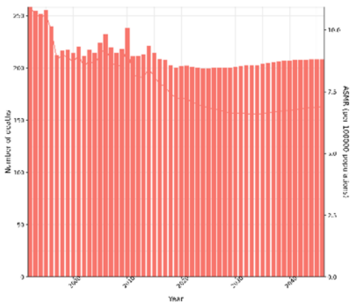

Romania

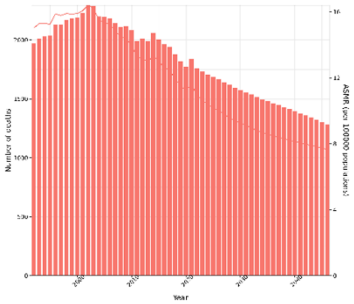

Russian Federation

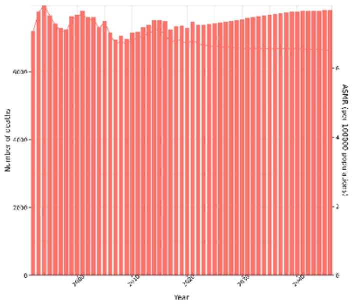

Serbia

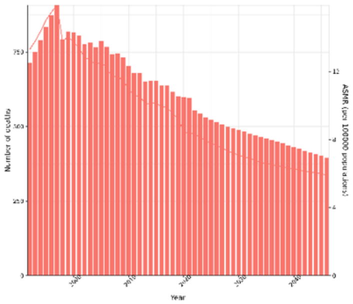

Slovakia

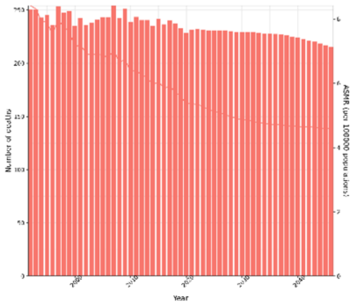

Slovenia

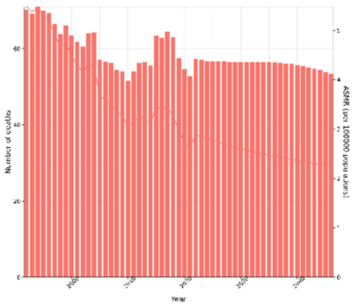

Spain

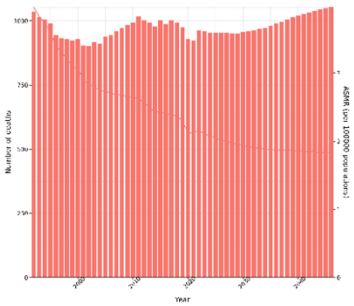

Sweden

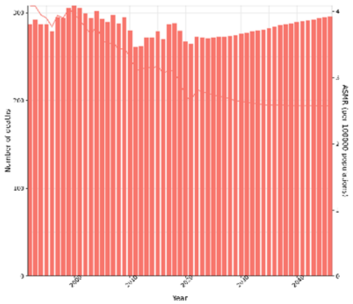

Switzerland

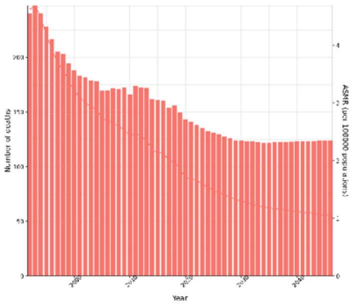

Ukraine

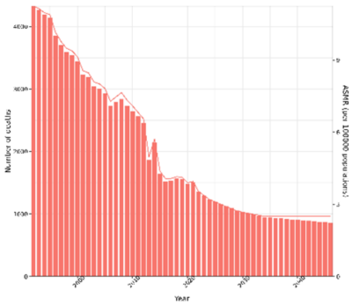

United Kingdom

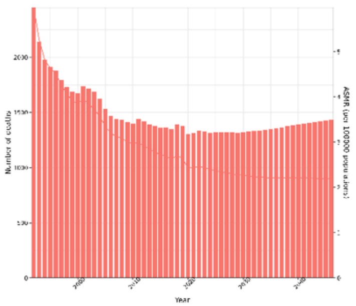

Figure S22 (Continue). Observed and predicted cervical cancer-related death numbers and age-standardized mortality rate in 44 countries within the European Region from 1992 to 2046.
